# Calcium-rich dairy matrix protects better than mineral calcium against colonic luminal haem-induced alterations in male rats

**DOI:** 10.1038/s41538-024-00273-y

**Published:** 2024-07-02

**Authors:** Maïwenn Olier, Nathalie Naud, Edwin Fouché, Valérie Tondereau, Ingrid Ahn, Nadine Leconte, Florence Blas-Y-Estrada, Gilles Garric, Cécile Heliès-Toussaint, Marielle Harel-Oger, Corinne Marmonier, Vassilia Théodorou, Françoise Guéraud, Gwénaël Jan, Fabrice Pierre

**Affiliations:** 1 Toxalim (Research Centre in Food Toxicology), INRAE, Université de Toulouse, ENVT, INP-EI Purpan, UPS, Toulouse, France; 2 STLO, INRAE, I’Institut Agro, Rennes, France; 3 CNIEL, Paris, France

**Keywords:** Gastrointestinal diseases, Physiology

## Abstract

The haemoglobin content in meat is consistently associated with an increased risk of colorectal cancer, whereas calcium may play a role as a chemopreventive agent. Using rodent models, calcium salts have been shown to prevent the promotion of haem-induced and red meat-induced colorectal carcinogenesis by limiting the bioavailability of the gut luminal haem iron. Therefore, this study aimed to compare impacts of dietary calcium provided as calcium salts or dairy matrix on gut homoeostasis perturbations by high haeminic or non-haeminic iron intakes. A 3-week intervention study was conducted using Fischer 344 rats. Compared to the ferric citrate-enriched diet, the haemoglobin-enriched diet led to increased faecal, mucosal, and urinary lipoperoxidation-related biomarkers, resulting from higher gut luminal haem iron bioavailability. This redox imbalance was associated to a dysbiosis of faecal microbiota. The addition of calcium to haemoglobin-enriched diets limited haem iron bioavailability and counteracted redox imbalance, with improved preventive efficacy when calcium was provided in dairy matrix. Data integration revealed correlations between haem-induced lipoperoxidation products and bacterial communities belonging to *Peptococcaceae*, *Eubacterium coprostanoligenes* group, and *Bifidobacteriaceae*. This integrated approach provides evidence of the benefits of dairy matrix as a dietary calcium vehicle to counteract the deleterious side-effects of meat consumption.

## Introduction

Based on systematic review of epidemiological studies, the latest revised World Cancer Research Fund (WCRF) and American Institute for Cancer Research (AICR) has reinforced the convincing evidence that, among the many drivers contributing to the incidence of colorectal cancer (CRC), lifestyle and eating habits are key modifiable risk factors^1^. Based on dietary surveys and probabilistic models, the International Agency for Research on Cancer (IARC) proposed in 2018 that about 5% of CRC cases would be attributable to red meat consumption^2^. Dose–response meta-analyses have also consistently shown an increased risk of colon cancer with increased consumption of red meat, leading IARC to conclude that red meat is probably carcinogenic to human (group 2A)^3^, and WCRF to recommend limiting its consumption (<500 g/week)^4^. Despite these recommendations, 33–40% of men and 9–20% of women would exceed these amounts^5–7^.

Among the red meat constituents susceptible to contribute to CRC, both epidemiologic and experimental evidence support a role of dietary haemoglobin through its haem iron content^8–12^. Mechanistically, when it reaches the colon, haem iron, as compared to non-haem iron, is suspected to mediate a luminal deleterious environment through its ability to catalyse lipid oxidation of dietary polyunsaturated fatty acids^13,14^. This oxidative process leads to the neoformation of secondary reactive end products, of which aldehydes such as 4-hydroxy-2-nonenal (4-HNE) are the most prominent, and known to be associated with free radicals release, cytotoxicity, genotoxicity, gut epithelial barrier defect and preneoplastic lesions in rodents^14–20^. In addition, both haem bioavailability and increase of haem-induced lipid oxidation products in the colon have been shown to be microbiota-dependent, and closely correlated with a microbiota reshape^14,21,22^.

In vitro studies have shown that calcium salts (calcium phosphate, calcium carbonate…), but not calcium ions, induce the precipitation of a solution of bilirubin, haemin and haemoglobin^23^. In vivo, while haem iron concentration in faeces is not altered by the addition of dietary calcium salts, the latter limits the solubilisation of haem iron in faecal water, and therefore limit its luminal bioavailability in the colon^11,14,23–25^. Consequently, neoformation of lipoperoxidation products and related-microbiota dysbiosis were alleviated, resulting in prevention of pathophysiological features, including the promotion of carcinogenesis^11,14,24^.

Dairy products are calcium-dense foods, especially cheese but also fermented milk like yogurts, and are widely consumed globally^26^. They contribute significantly to calcium intake for humans^27,28^. Since previous studies were carried out with calcium salt supplementation, the question arises whether calcium provided in a dairy matrix would have the same efficacy. Therefore, the present animal study was designed to assess whether the prevention of a haem-driven deleterious colonic environment by dietary calcium intake could depend on its origin, i.e. as mineral salt supplementation or as dairy matrix. To this aim, we compared the cross-impacts of the iron form, haem (provided as haemoglobin) vs. non-haem (provided as ferric citrate), on gut homoeostasis and peroxidative stress biomarkers in rats, according to dietary calcium supplementation provided by mineral salt (Ca-phosphate) or a dairy matrix. Given the contribution of the gut microbiota in haem-induced lipoperoxidation, modulations of its structure and composition in response to dietary iron and calcium contents were analysed.

## Results

### Impact of dietary calcium intake on luminal haem iron bioavailability and haem-induced lipid peroxidation-related biomarkers

**Fig. 1:**
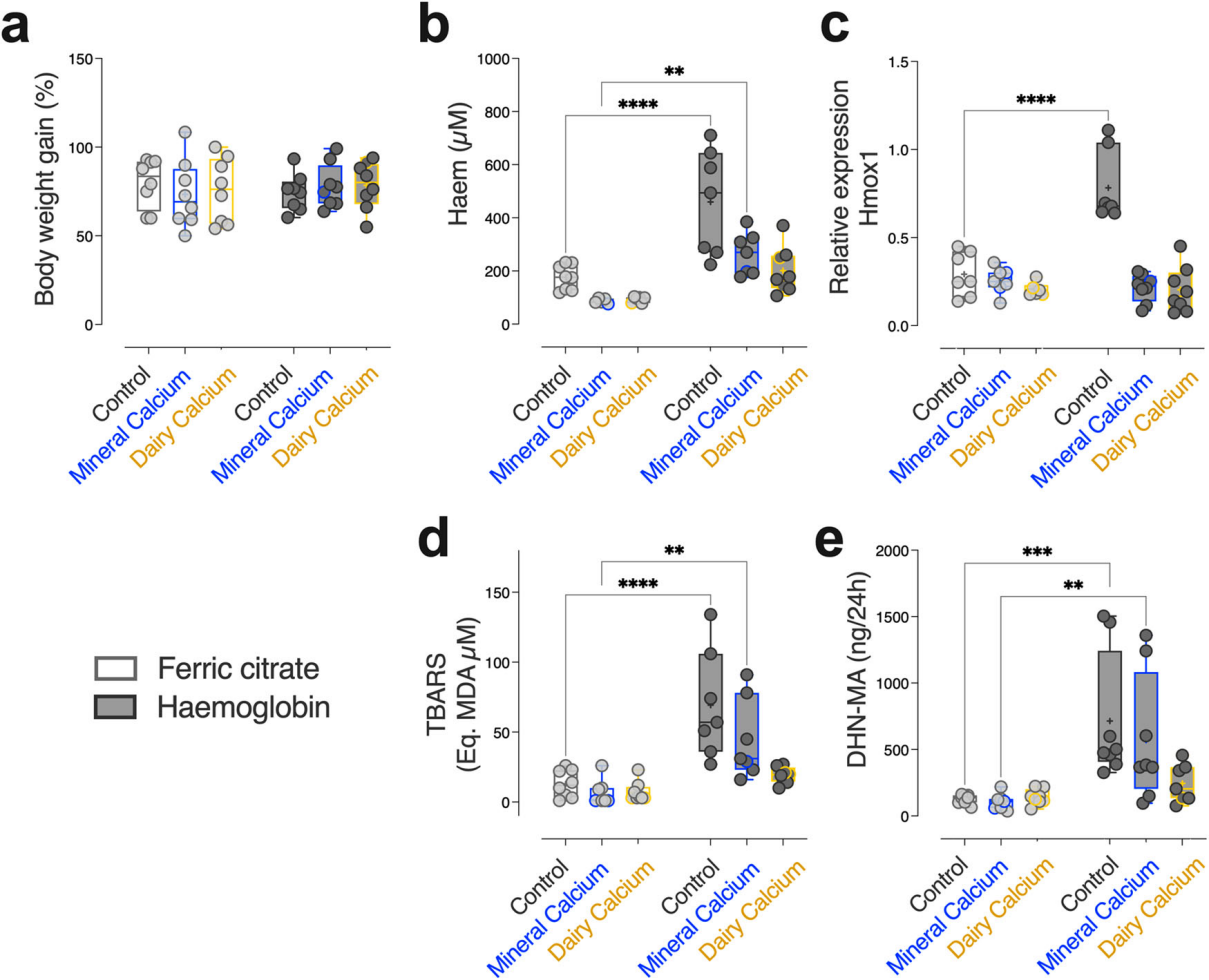
Physiological and peroxidative stress parameters monitored in Fischer 344 rats after 23 days of dietary intervention (*n* = 48). Measurements of bodyweight gain (**a**), faecal haem (**b**), colonic expression of haem oxygenase 1 (**c**), faecal TBARS (**d**) and urinary DHN-MA (**e**) according to dietary iron (Ferric citrate vs. Haemoglobin) and calcium (Control vs. Mineral vs. Dairy) contents. Individual values within box and whisker are represented with “+” as means. For statistical analysis, two-way ANOVA, followed by Holm-Šídák’s multiple comparisons tests were performed. **p_*adj*_ < 0.01, ***p_*adj*_ < 0.001, ****p_*adj*_ < 0.0001 according to dietary iron form. Detailed *F*-statistic and *p* values are provided as Supporting information in Table S1.

During the 3-week dietary intervention, the rats’ body weight gain was not affected by the diet composition (Fig. 1a). As expected, in rats fed the Control diets (low calcium content), the addition of haemoglobin significantly increased the haem levels detected in faecal waters, when compared to the addition of ferric citrate (Fig. 1b, *F*_iron_ = 36.9, *p*_*iron*_ and *p*_*adj Control*_ < 0.0001). Increase in faecal haem content in this group (haemoglobin-supplemented Control diet) was also associated with an increased mucosal expression of the Haem Oxygenase-1gene (Hmox1, Fig. 1c, *F*_iron_ = 16.2, *p*_*iron=*_0.003 and *p*_*adj Control*_ < 0.0001), and an increased neoformation of faecal and urinary lipid peroxidation biomarkers, as revealed by the measurements of TBARS (Fig. 1d, *F*_iron_ = 34.7, *pi*_*ron*_ and *p*_*adj Control*_ < 0.0001) and DHN-MA (Fig. 1e, *F*_iron_ = 20.1, *p*_*iron*_ < 0.001 and *p*_*adj Control*_ = 0.007) respectively.

Regardless of the form of the iron in diets, addition of dietary calcium, through its known trapping properties, is supposed to reduce the bioavailability of haem during the transit at the luminal side^20^. As such, lower detection of haem in faecal waters was indeed observed regardless of the calcium source (mineral or dairy, Fig. 1b, *F*_*calcium* _= 12.2, *p*_*calcium*_ < 0.0001, Supporting Information Table S1). Interestingly however, the efficiency of haem trapping by dietary calcium supplementation was higher when it was derived from the dairy matrix rather than from the mineral origin (haemoglobin *vs*. ferric citrate, *p*_*adj MineralCa*_ = 0.0081 in mineral calcium-enriched diets, *p*_*adj DairyCa*_ = 0.05 in dairy matrix-enriched diets). Similarly, reduced haem bioavailability resulting from calcium addition to haemoglobin-enriched diets was associated with the absence of haem-induction of Hmox1 gene expression (Fig. 1c, *F*_*calcium* _= 32.6, *p*_*calcium*_ < 0.0001, haemoglobin *vs*. ferric citrate *p*_*adj Mineral/Dairy*_ > 0.05 in diets supplemented with mineral or dairy calcium). Compared with diets containing ferric citrate, the addition of dietary calcium to haemoglobin-enriched diets limited the neoformation of faecal (TBARS) and urinary (DHN-MA) lipoperoxidation biomarkers, and even prevented their formation when calcium was provided by the dairy matrix (Fig. 1d, e, haemoglobin vs. ferric citrate, *p*_*adj MineralCa*_ < 0.001 in mineral calcium-enriched diets, *p*_*adj DairyCa*_ > 0.05 in dairy matrix-enriched diets).

### Cross-impacts of dietary haemoglobin and calcium intake on gut homoeostasis

**Fig. 2:**
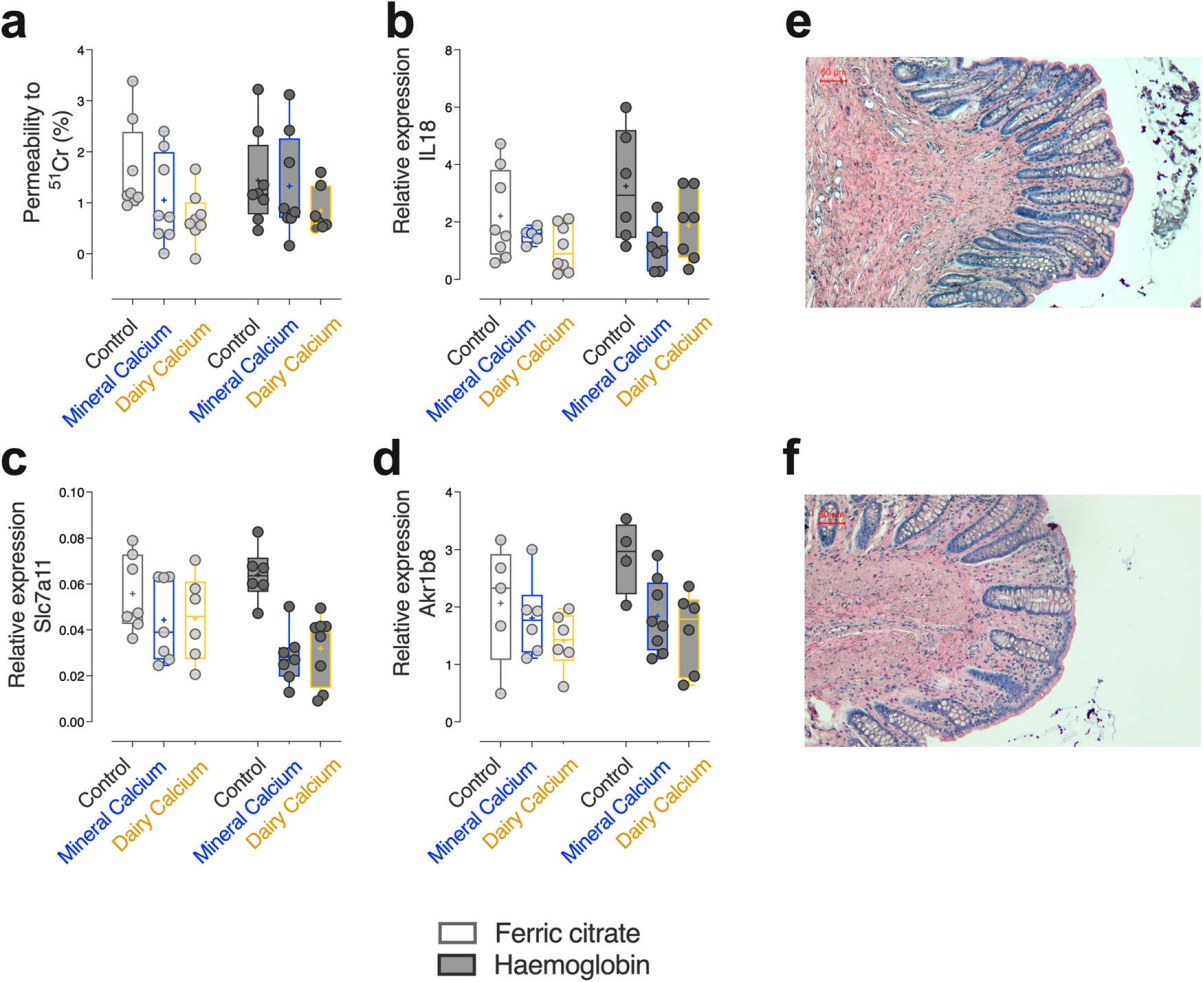
Gut barrier homoeostasis evaluation in Fischer 344 rats after 23 days of dietary intervention (*n* = 48). Percentage of total radioactivity excreted in urines 24 h after oral administration of ^51^Cr (**a**), mucosal relative gene expression of IL-18 (**b**), Slc7a11 (**c**) and Akr1b8 (**d**) by qPCR analysis according to dietary iron (Ferric citrate vs. Haemoglobin) and calcium (Control *vs*. Mineral vs. Dairy) contents. Representative histological images of colonic mucosa of rat fed a control diet supplemented with (**e**) ferric citrate and (**f**) haemoglobin. Scale bar, 60 µm. Individual values within box and whisker are represented with “+” as means. For statistical analysis, two-way ANOVA, followed by Holm-Šídák’s multiple comparisons tests were performed. **p_*adj*_ < 0.01, ***p_*adj*_ < 0.001, ****p_*adj*_ < 0.0001 according to dietary iron form. Detailed *F*-statistic and *p* values and relative expressions are provided as Supporting Information Tables S1, S2.

Compared with diets containing ferric citrate, haemoglobin-enriched diets did not significantly alter barrier function in terms of paracellular permeability to ^51^Cr (Fig. 2a), nor the mucosal gene expression related to inflammation (Fig. 2b), detoxification pathways (Fig. 2c, d) (*F*_iron _< 2, *p*_*iron*_ > 0.05, Supporting Information Table S1, S2). No particular histological damage was observed within colonic crypt architecture according to dietary iron (Fig. 2e, f). Regardless of the iron form in diets however, the addition of dietary calcium led to an enhanced intestinal barrier function associated with reduced expression of the genes related to inflammation and aldehyde detoxification (*F*_calcium_ >3.4, *p*_*calcium*_ < 0.05 in a, b, and d, *F*_*calcium*_ = 9.3, *p*_*calcium*_ < 0.01 in c, Supporting Information Table S1). This improvement in presence of calcium was particularly noticeable, although not significant, when calcium was provided by the dairy matrix compared to the mineral one.

### Faecal microbiota structure and composition according to iron and calcium contents

**Fig. 3:**
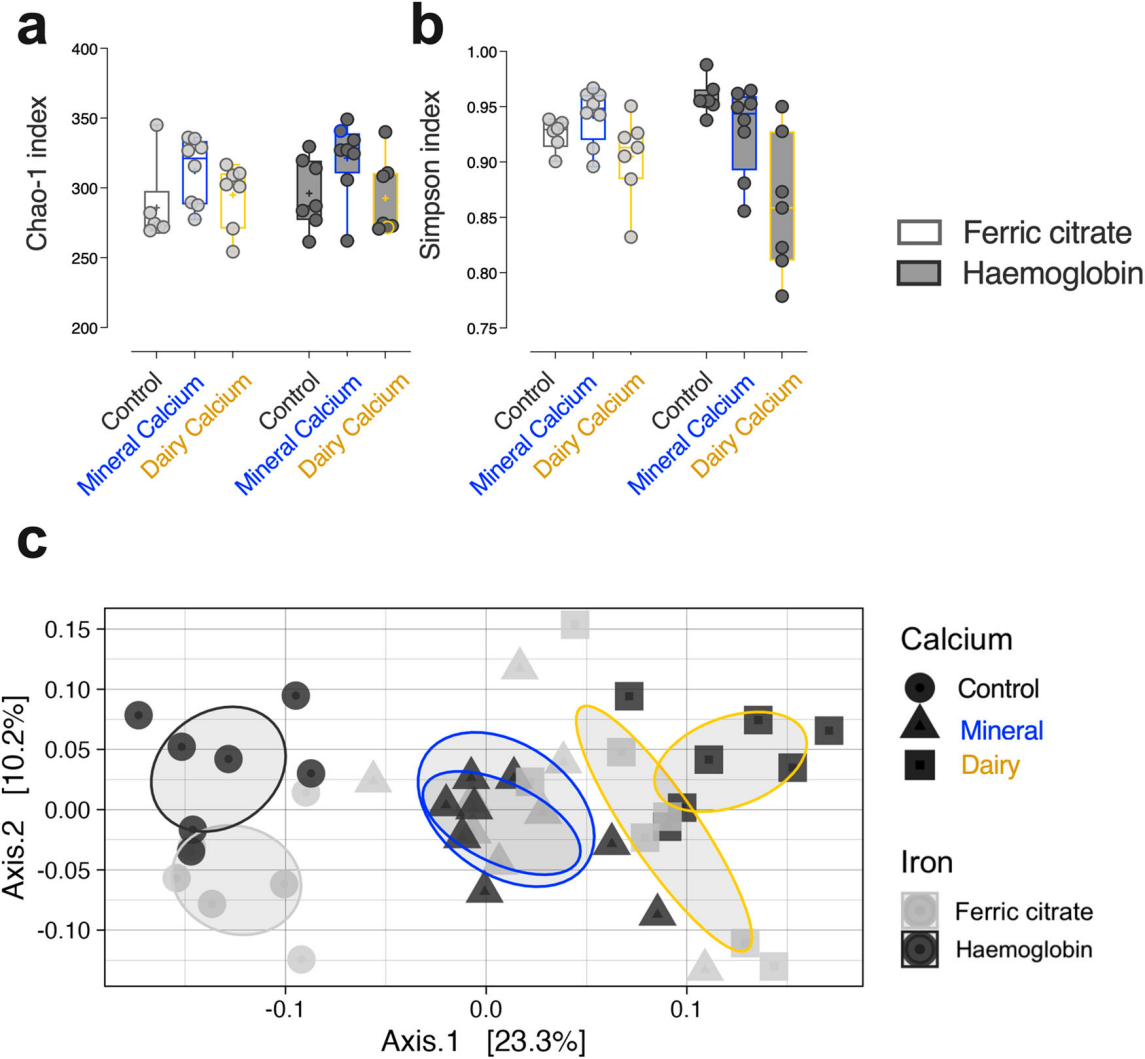
Faecal microbiota diversity changes after dietary intervention (*n* = 43). *α*-diversity indices were assessed using Chao-1 (**a**) and Simpson (**b**) estimators for richness and evenness assessment respectively. *β*-diversity was explored using Unifrac distances and multidimensional scaling (**c**). Individual values according to dietary iron (Ferric citrate vs. Haemoglobin) and calcium (Control vs. Mineral vs. Dairy) contents are plotted. Statistical support for differences between samples across diets was obtained by Adonis test with 9999 permutations followed by pairwise multilevel comparison. Detailed *F*-statistics and *p* values are provided as Supporting Information Table S1.

In order to assess *α*-diversity of the faecal microbiota according to diets, both richness and evenness were determined using the Chao-1 index and Simpson index, respectively (Fig. 3a, b). None of these indices were affected by the iron form (*F*_*iron*_ = 0.5, *p*_*iron*_ > 0.05), but they were modulated by both the dose and origin of dietary calcium (*F*_*calcium*_ = 4.5, *p*_*calcium*_ < 0.05 in a, *F*_*calcium*_ = 11, *p*_*calcium*_ < 0.01 in b, Supporting Information Table S1). Greater microbiota richness was effectively associated with a higher intake of calcium of mineral origin, and lower microbiota evenness was associated with intake of calcium of dairy origin.

Exploration of *β*-diversity using Unifrac distance matrices revealed a clear separation of rat faecal microbiota structure along Axis.1 based mainly on the quantity and origin of calcium intake (Fig. 3c, *F*_*calcium*_ = 7.3, *p*_*calcium*_ < 0.0001). Axis.2 significantly separates rats fed a ferric citrate-enriched Control diet from those fed the haemoglobin-enriched Control diet (*p*_*adj Control*_ = 0.01), whereas this iron-dependent separation was reduced, if not lost, in rats fed diets enriched with calcium regardless of its origin (*p*_*adj Mineral/Dairy*_ > 0.05).

**Fig. 4:**
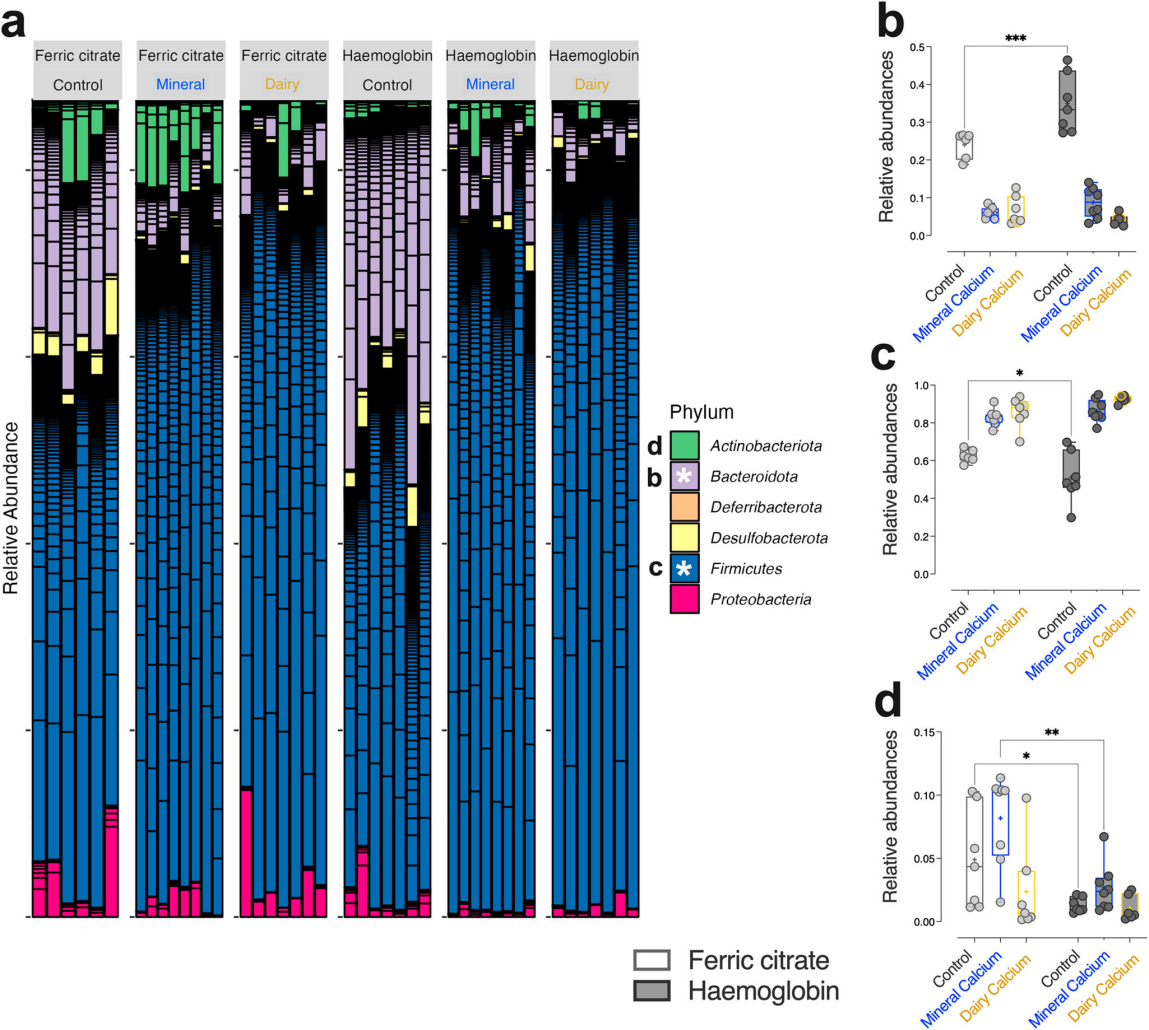
Relative faecal microbiota distribution changes after dietary intervention (*n* = 43) at the phylum level. **a** Relative abundances (%) of *Bacteroidota* (**b**), *Firmicutes* (**c**) and *Actinobacteriota* (**d**) according to dietary iron (Ferric citrate vs. Haemoglobin) and calcium (Control vs. Mineral vs. Dairy) contents are detailed. Individual values within box and whisker are represented with “+” as means. For statistical analysis, two-way ANOVA, followed by Holm-Šídák’s multiple comparisons tests were performed. *p_*adj*_ < 0.05, **p_*adj*_ < 0.01, ***p_*adj*_ < 0.001 according to dietary iron form. Detailed *F*-statistic and *p* values are provided as Supporting Information Table S1.

In accordance with *β*-diversity results, phyla distribution is mainly influenced by dietary calcium content (Fig. 4), as also suggested by the comparison of the number of discriminative bacterial features revealed by Linear discriminant analysis Effect Size (LEfSe) according to diets (Supporting information Figs. S1, S2). Indeed, depending on whether the calcium or iron factor is considered, an average of 140 bacterial features were significantly affected by the supplementation of dietary calcium at *p* < 0.01 (Fig. S1a, b, d, e), whereas only an average of 44 bacterial features were significantly affected by the dietary iron form used at *p* < 0.05 (Fig. S2), making identification of bacterial taxa modulated by the both factors difficult. Still, of the 6 phyla detected, the first 3 most prevalent ones, *Bacteroidota* (Fig. 4a, b), *Firmicutes* (Fig. 4a, c) and *Actinobacteroidota* (Fig. 4a, d) were modulated by both the iron form and calcium content, knowing that for *Bacteroidota* and *Firmicutes*, the effect of iron was calcium-dependent (*F*_*iron* × *calcium*_ > 6, *p*_*iron* *×* *calcium*_ < 0.01, Supporting information Table S1): Indeed, in both cases, significant changes in relative abundance in response to haemoglobin-enriched Control diet *vs*. the ferric citrate-enriched Control diet were alleviated by the addition of dietary calcium, regardless of its origin.

**Fig. 5:**
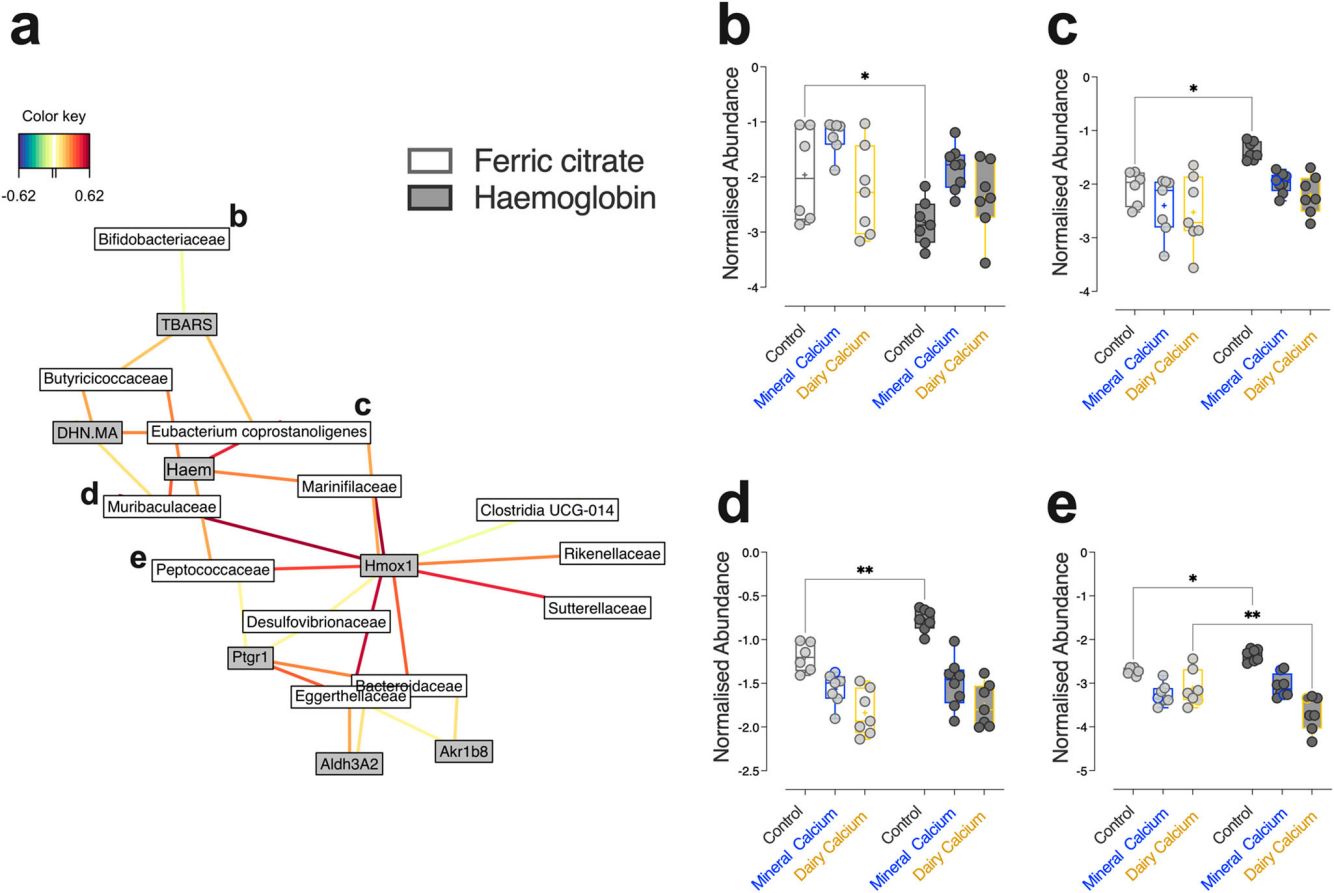
Network plot resulting from PLS representing correlations between normalised abundances of OTUs agglomerated at the family level and physiological biomarkers monitored in 42 rats. **a** Only correlations higher than 0.62 are represented. Red and blue colours indicate positive and negative correlation respectively. Normalised abundances of *Bifidobacteriaceae* (**b**), *Eubacterium coprostanoligenes* (**c**) and *Muribaculaceae* (**d**) and *Peptococcaceae* (**e**) according to dietary iron (Ferric citrate *vs*. Haemoglobin) and calcium (Control vs. Mineral vs. Dairy) contents are detailed. Individual values within box and whisker are represented with “+” as means. For statistical analysis, two-way ANOVA, followed by Holm-Šídák’s multiple comparisons tests were performed. *p_*adj*_ < 0.05, **p_*adj*_ < 0.01, ***p_*adj*_ < 0.001 according to dietary iron form. Detailed *F*-statistic and *p* values are provided as Supporting Information Table S1.

In order to directly identify the bacterial families whose normalised abundances most covaried with each of the previously monitored biomarkers, a PLS allowing datasets integration (46 bacterial families and 38 physiological parameters monitored in 42 rats) was performed. The network plot showing correlations higher than 0.62 (Fig. 5a) revealed that the highest positive ones linked the biomarkers related to bioavailable luminal haem (Haem, Hmox1, DHN-MA and TBARS) to bacterial communities belonging to the group of *Eubacterium coprostanoligenes, Muribaculaceae*, and *Peptococcaceae*, whereas normalised abundances of *Bifidobacteriaceae* were negatively correlated with TBARS levels. For these families (Fig. 5b–e), significant changes in normalised abundance in response to the haemoglobin-enriched Control diet vs. the ferric citrate-enriched one was alleviated by the addition of dietary calcium, regardless of its origin.

**Fig. 6:**
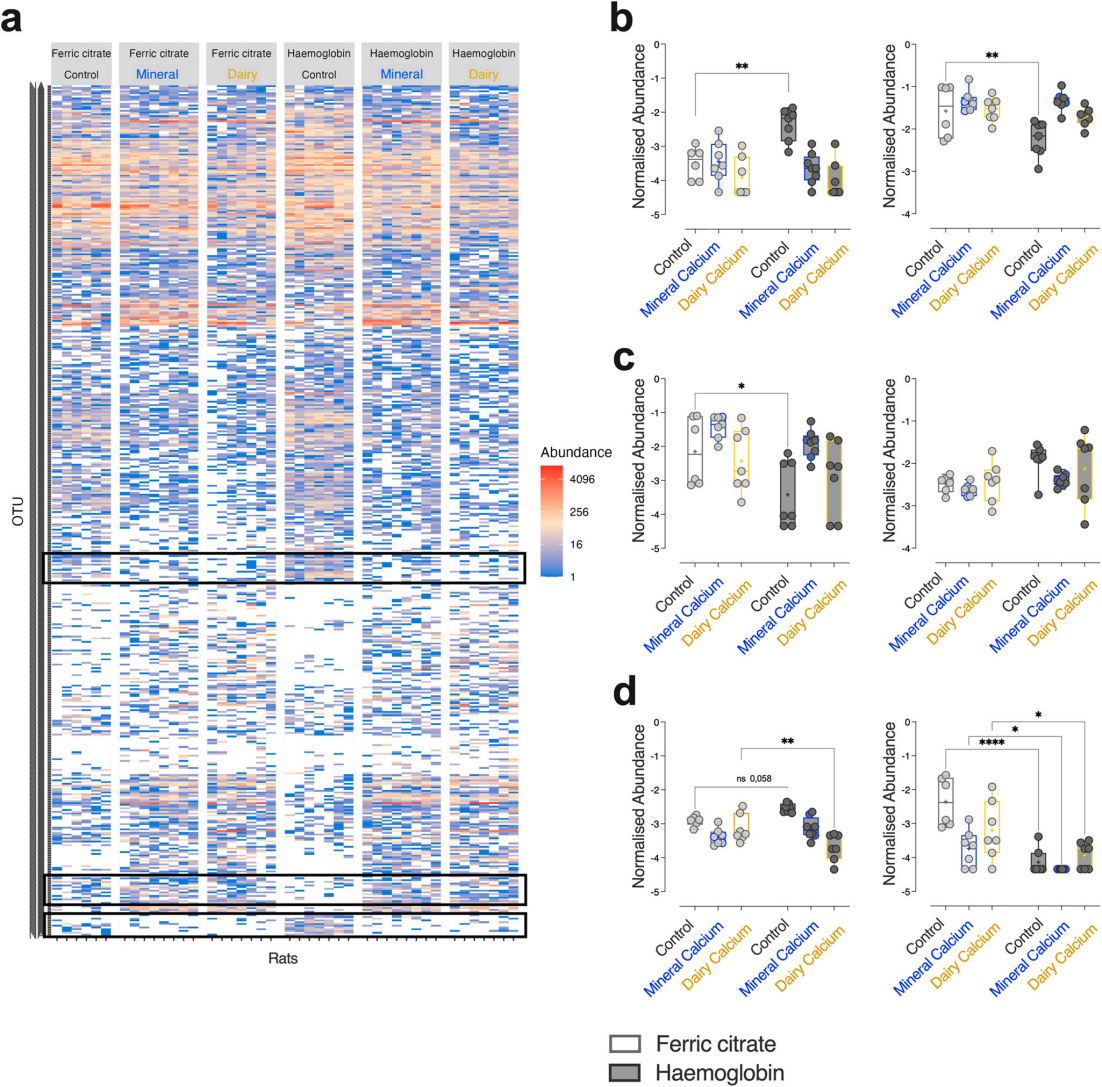
Prevalence of OTUs in 43 rats according to dietary iron (Ferric citrate vs. haemoglobin) and calcium (control vs. mineral vs. dairy). **a** The heatmap represents the abundance of the 486 OTUs detected in each rat (1 rat per column) and clusters of OTUs boxed in black correspond to OTUs whose changes induced by the addition of haemoglobin *vs*. ferric citrate are normalised by the addition of calcium, (**b**) regardless of its origin (*Eisenbergiella spp*., multi-affiliated *Lachnospiraceae* among others), (**c**) preferentially when it is from mineral origin (*Bifidobacterium pseudolongum*, *Lachnoclostridium* among others) or (**d**) from dairy origin (unknown *Peptococcaceae*, *Lactobacillus intestinalis* among others). For (**b**, **c**, **d**), abundances were obtained once OTUs were agglomerated at the species or genus levels. Individual normalised values were plotted within the box and whisker with “+” as means according to dietary iron (Ferric citrate *vs*. Haemoglobin) and calcium (Control vs. Mineral vs. Dairy) contents. For statistical analysis, two-way ANOVA, followed by Holm-Šídák’s multiple comparisons tests were performed. *p_*adj*_ < 0.05, **p_*adj*_ < 0.01, ****p_*adj*_ < 0.0001 according to dietary iron form. Detailed *F*-statistic and *p* values are provided as Supporting Information Table S1.

By performing similar approaches at a finer scale (Fig. 6), clusters of OTUs of interest were targeted (Fig. 6a) and corresponded notably once agglomerated at the species/genus levels to bacterial communities differentially altered by dietary haemoglobin according to the dietary calcium content (Fig. 6b–d, *F*_*iron*_ and/or *F*_*calcium*_ and/or *F*_*iron* x calcium_ > 3.2, *p*_*iron*_ and/or *p*_*calcium*_ and/or *p*_*iron* *×* *calcium*_ $\le$0.05). Among them, several scenarios were indeed emerged towards dietary calcium supplementation: haemoglobin-altered abundances of *Eisenbergiella spp*. and multi-affiliated *Lachnospiraceae* notably (haemoglobin vs. ferric citrate *p*_*adj Control*_ < 0.01 in Control diets) returned to levels observed with ferric citrate-enriched diets, regardless of the calcium origin (Fig. 6b, haemoglobin vs. ferric citrate *p*_*adj Mineral/Dairy*_ > 0.05 in diets supplemented with mineral or dairy calcium). In the case of *Bifidobacterium pseudolongum* or *Lachnoclostridium*, haemoglobin-altered abundances were normalised by dietary calcium intake preferentially when provided by the mineral origin (Fig. 6c), whereas in the case of unknown *Peptococcaceae* or *Lactobacillus intestinalis*, normalisation was particularly noticeable when calcium was of dairy origin (Fig. 6d).

In parallel, for some haemoglobin-altered bacterial communities, dietary calcium intake did not restore abundances to levels similar to those observed in rats fed the ferric citrate-enriched Control diet (Fig. S3). Conversely, for many other bacterial communities such as *Desulfovibrio spp*., some unclassified *Enteroccocus*, and *Negativibacillus spp*., the dietary haemoglobin did not alter their abundance, but interestingly, the dietary calcium intake differentially altered their abundance depending on its quantity and/or origin (Fig. S4a-b). Finally, some bacterial communities, such as *Frisingicoccus caecimuris*, *Macrococcus spp*. and *Exigobacterium spp*. were exclusively found in the faeces of rats fed diets supplemented with the dairy matrix and were not influenced by the iron form used (Fig. S4c).

## Discussion

In an aim to provide further evidence on how the combination of individual dietary micro-nutrients may modulate CRC risk, we conducted a short-term nutritional experiment in rats to compare the biological interrelations between iron and calcium according to their source, by measuring (i) the luminal haem bioavailability, and (ii) haem-related early related biomarkers known to contribute to haem-induced carcinogenesis promotion. Our results revealed that specific gut bacterial populations modulations elicited by haem were strongly dependent on both the calcium level and its source, and strongly correlated with gut luminal lipoperoxidation status, revealing in summary, a stronger effect of calcium intake in the form of dairy matrix by limiting haem intestinal bioavailability and subsequent haem-induced luminal peroxidation.

In line with our previous studies^14,24^, the luminal increase in free haem iron, resulting in this experiment from intake of haemoglobin vs. ferric citrate, induced a pro-oxidative colonic environment, as evidenced by Hmox1 expression, and the neoformation of lipoperoxidation products. In the same way, DHN-MA levels in urine attested that part of the lipoperoxidation products detected in faecal water such as 4-HNE, were absorbed and metabolised by the epithelium. However, on the mucosal side, in contrary to what we observed in the study of ref. ^14^, lipoperoxidation induced by the replacement of ferric citrate by haemoglobin in the diet was not associated with gut barrier defects, as revealed by permeability measurement and inflammation- and detoxification-related gene expression. The use of haemoglobin herein, instead of hemin used in the study of Martin et al, may explain this lack of effect on the colonic mucosa. Indeed, Pierre et al. previously demonstrated that at equimolar amount of haem in the diet, the free haem detected in faecal water were slightly lower with dietary haemoglobin than with hemin, resulting in a lower promotion of lipid peroxidation and, consequently, associated with lower cytotoxicity and fewer preneoplastic lesions in rats^11^. In addition to the form of haem iron used in the diet, a lower haem content was used in the present study (0.15 µmol/g haem) as compared to the dose used by Martin et al. (1.5 µmol/g haem provided by haemin, knowing that 1.5 µmol of haemin has the same haem content as 0.36 µmol haemoglobin)^14^. Probably in relation to the dose and/or the form of luminal colonic exposure to haem iron, the alteration of microbiota in response to haemoglobin also differed from that previously described with haemin exposure^14,22^. While haemin intake deeply reduced the alpha diversity indices^14^, no change in richness or evenness was observed in our study. Beta diversity was significantly shifted in both cases, but resulted in variations in some bacterial communities depending on the luminal haem source. Indeed, at the phylum level, the major increase of *Bacteroidota* at the detriment of *Firmicutes* was maintained, but the *Proteobacteria* increase mainly attributed to the *E. coli* bloom was no longer observed, and the decrease of *Actinobacteria* mainly attributed to *Bifidobacteria* in this study seems to be specific to haemoglobin exposure.

Independently or not of the haem-mediated oxidative stress, existing literature has already demonstrated the protective role of dietary calcium against food-borne infection^29^ and microbiota disruption^14,30^, gut barrier defect^14,31,32^, oxidative and inflammatory stress^14,33^. Furthermore, total dairy products, certain sub-categories (milk, cheese and yoghurts) as well as dairy calcium has been shown to prevent CRC using meta-analysis of epidemiological studies as well as rodents models^24,34–38^. Among the possible mechanisms underlying the calcium protective effect, activation of epithelial calcium-sensing receptors leading to intracellular adaptations, such as gut barrier permeability and cytokines regulation, has been described^39,40^. However, this calcium-dependent signalling process does not appear to be sufficient to explain the observed luminal changes. Regarding the luminal side, calcium is also reported to bind to and to precipitate with free ionised long-chain fatty and bile acids, thereby contributing to reduce their solubility in the luminal environment and their toxicity toward the mucosal barrier^41^. In agreement with previous study, our findings confirmed that mucosal barrier integrity, although it was not impacted by dietary haemoglobin, was improved by dietary calcium^14^. Regarding the interplay between haem content from dietary haemoglobin and mineral calcium salts within the soluble fraction (faecal water), bioavailability of haem, as previously observed, was reduced by calcium phosphate intake, and resulted in limitation of lipoperoxidation products neoformation^11,23,24^.

Interestingly, comparison of dietary calcium source, mineral vs. dairy, revealed that haem trapping was more efficient with calcium in dairy matrix, even resulting in normalisation of levels of luminal lipoperoxidation products when compared with those resulting from non-haem iron (ferric citrate). Consistent with this, Zemel and Sun reported that, compared to dietary calcium in the Ca-carbonate form, dairy products were more effective in inhibiting obesogenic diet-induced plasma lipid peroxidation in mice^33^. However, the reduction in oxidative stress observed at the systemic level in their study may also be explained by the associated reduction in weight gain and fat mass. Relatedly, the incidence of tumours in azoxymethane-treated rats was reported to be decreased by a high-fat diet supplemented with nonfat dried milk compared with Ca-carbonate^35^. These results are also consistent with some of the data in the literature and the conclusion of the meta-analysis performed by Emami et al., that suggest that calcium of dairy products was more effective than supplementary calcium to limit the risk of colorectal adenomas and advanced adenomas^36^. However, if WCRF suggested that calcium may prevent haem-induced promotion of colon carcinogenesis^1^, the interaction of calcium with iron, and in particular haem iron, has not been considered by Emami et al. Our results may contribute to explain the calcium enhanced protection observed against CRC when sourced from food and especially dairy products. However, further studies are needed to identify the underlying mechanisms, and to verify whether additional components of the dairy matrix do not contribute to this effect. Indeed, the nature of proteins and bioactive peptides found in dairy matrix, due to their antioxidant properties and/or iron complexation^42,43^, may explain the improved prevention of haem-induced lipoperoxidation observed in our study. It is also conceivable that an enhanced luminal bioavailability of dietary calcium when sourced from dairy matrix, due to the formation of casein phosphopeptides^44^, may explain its greater ability to reduce haem bioavailability, and thus alleviate haem-induced lipoperoxidation.

The modulation of gut microbiota composition according to calcium source and its contribution to the abrogation of haem-induced lipoperoxidation are also to be considered. As previously reported using Ca-carbonate and haem iron^14^, strong correlations between luminal haem iron bioavailability (modulated by dietary calcium), lipoperoxidation status, and gut bacterial communities were reproduced. These correlations confirm a protective effect of dietary calcium intake, regardless of its origin, against dysbiosis associated with haem-induced lipoperoxidation.

Among the more significant changes induced by calcium supplementation in our study, we can mention the (i) improvement of haemoglobin-altered commensal communities abundance belonging to *Bifidobacteriaceae* or *Lactobacillus intestinalis*, *i.e*. communities whose health benefits, partly due to their antioxidant properties, have been previously reported^45,46^, and (ii) the reduction of *Peptococcaceae* or *Lachnoclostridium*, i.e. pathobionts previously proposed as faecal or salivary biomarkers in the panel of tools allowing the diagnosis of colorectal adenomas and cancers^47–50^. For some of them however, dietary calcium influenced abundances in an origin-dependent manner: calcium of mineral origin more effectively normalised *Bifidobacterium pseudolongum* and *Lachnoclostridium* abundances, while calcium of dairy origin more specifically counteracted the haem-induced increase in taxa belonging to *Peptococacceae* and *Lactobacillus intestinalis*. Given this highly variable adaptative bacterial behaviour from one community to another depending on the dose and source of calcium encountered, we can presume that, beyond the form of iron encountered by bacteria, the main underlying drivers that may determine gut microbiota reshape are the resulting degree of luminal iron bioavailability and, consequently, (i) the variable aptitude of gut bacteria for dealing with it to meet their own iron requirements, and (ii) the variable aptitude of gut bacteria to avoid iron-mediated oxidative stress. Indeed, depending on their metabolism (mode of respiration), their structural envelope (Gram-negative/positive) and their genetic equipment (encoding a myriad of systems including siderophores, haemophores.), bacteria may or may not be able to sense the iron status and regulate pathways involved in the acquisition and storage of iron accordingly. With regard to their variable aptitude to cope with the pro-oxidative environment encountered, or even to reduce the oxidative burden, the vast majority of work investigating such fitness factors has focused on pathogens^51,52^. Nevertheless, comparative genomic analyses have shown that some gut commensals are also equipped (superoxide dismutases or reductases, PerR, rubrerythrins.)^53,54^, and measurements of antioxidative potential among *Bifidobacteriaceae* and lactic acid bacteria have revealed the ability of certain strains to inhibit lipoperoxidation in vivo and in vitro using liposomal suspensions^55^.

In summary, this experimental study shows that dietary calcium provided by the dairy matrix results in a greater reduction of the luminal haem iron bioavailability and, consequently, greater protection against haemoglobin-induced lipoperoxidation compared to the intake of mineral calcium. Our data exemplifies the complexity of the luminal interplay between micronutrients such as iron and calcium depending on their origin, in terms of form and source in food. This study also shows how this interplay can condition the bioavailability of haem iron, steer the dynamics of bacterial communities, and the associated homoeostasis of the gut luminal environment. Understanding how the source of calcium intake can prevent excess lipoperoxidation is indeed an interesting approach to better adjust dietary recommendations to promote CRC prevention.

## Methods

### Experimental model and ethical approval

Male Fischer 344 rats (F344/DuCrl, *n* = 48) were purchased at 6 weeks from Charles River laboratories (Lyon, France). Rats were kept at a constant temperature of 22 °C on a 12-h light-dark cycle and fed their experimental diet ad libitum daily just before their active period. The experimental protocol was approved by the Animal Care Use Committee (Comité d’Ethique Pharmacologie-Toxicologie-Occitanie Toulouse, registered as no. 86 at the Ministry of Research) under the authorisation number [#16138-TOXCOM 214FP], and conducted in accordance with the European Union guidelines.

### Experimental diets

The experimental diets were all based on an AIN-76A diet provided by the Sciences de l’Animal & de l’Aliment Unit (INRAE, Jouy-en-Josas) and adapted to minimise dietary calcium content (0.09%). According to the composition of the diets described in Table 1, this semi-purified powdered-based diet was extemporaneously supplemented with different sources of iron (ferric citrate vs haemoglobin as proxy for red meat), lipids (provided as safflower oil and butter *vs* dairy matrix), and calcium (Ca-phosphate for the “Mineral Calcium” diet, vs dairy matrix for the “Dairy Calcium” diet) before being stored under vacuum at −20 °C. Except for calcium, all diets were balanced in iron, proteins, and lipids. The final dietary calcium contents were 22.5 µmol/g in the Control diets and 118 µmol/g in the Mineral/Dairy Calcium diets.

**Table 1:** Composition of experimental diets (g/100 g)

Groups	Ferric citrate			Haemoglobin		
	Control	Mineral Calcium	Dairy Calcium	Control	Mineral Calcium	Dairy Calcium
AIN-76 base diet low calcium, 20%casein	80.4	80.4	-	80.4	80.4	-
AIN-76 base low calcium, 5% casein	-	-	50	-	-	50
Ferric citrate	0.28	0.28	0.28	-	-	-
Haemoglobin	-	-	-	1	1	1
Safflower oil	5	5	5	5	5	5
Butter (82% fat)	14.4	14.4	-	14.4	14.4	-
(PO_4_)_2_Ca_3_	-	1.36	-	-	1.36	-
Dairy matrix	-	-	45	-	-	45

A dairy matrix was prepared at STLO (INRAE, Rennes) on a dedicated Dairy Platform. Bulk whole milk (250 kg) was purchased from Coopérative Sodiaal (Montauban de Bretagne, France). It was pasteurised (80 °C, 2 min) using a pilot Actijoule tubular electric exchanger (Actini, Maxilly-sur-Léman, France). Pasteurised milk was then concentrated by ultrafiltration on a pilot TIA/Pall ultrafiltration unit (TIA, Bollene, France) equipped with a Membralox P1960 ceramic membrane (19 channels, 6 mm in diameter, 1.02 m long, with a membrane pore size of 20 nm and a surface area of 1.08 m^2^, Pall Corporation, St Germain en Laye, France). Ultrafiltration was conducted at 50 °C as described previously^56^ until a 5.5-fold concentration of the retentate was achieved. Following UF treatment, retentate was collected in vats before cooling to 30 °C. Calcium phosphate was then added ((PO_4_)_2_Ca_3_, 21.71 g per kg of retentate), followed by rennet (extrait de présure Carlin, Danisco, France, 0.2 mL per kg of retentate). This modified retentate was then split into 320 g cheeses, which were cooled and stored at 4 °C. The gross composition of the cheese was as follows: pH 6.47, dry matter 43.58%, lipids 18%, proteins 15.03%, fat content in dry matter 48.18 and calcium 1.3%.

### Experimental design

Before the start of the dietary intervention, the rats were maintained in an acclimation phase for 7 days and fed a Control diet enriched with ferric citrate (Table 1). Then, rats were randomly allocated to six groups (*n* = 8) and assigned to experimental diets designed to assess the contribution of two dietary factors and their interaction using a 2 × 3 protocol: iron form (ferric citrate vs. haemoglobin) and calcium content/source (Control vs. Mineral Calcium vs. Dairy Calcium). During the dietary intervention (23 days), rat body weight as well as water and food intake measurements were recorded weekly. One week before sacrifice and tissue collection, 24-h faecal pellets and urine samples were collected from each rat housed in a metabolic cage to both quantify faecal and urinary biomarkers.

### Faecal and urinary peroxidative stress biomarkers

Faecal waters were prepared with 1 ml of distilled water and 50 µL of BHT in ethanol (butylated hydroxytoluene) for 0.42 g of dried frozen faeces as previously described^14^, and kept at −20 °C until use. Haem and thiobarbituric reactive subtances (TBARS) were measured in the faecal water according to ref. ^18^ and ^57^, respectively.

The urinary DHN-MA (1,4-dihydroxynonene mercapturic acid, a major urinary metabolite of 4-HNE) assay was performed using a competitive enzyme immunoassay as previously described^58^.

### In vivo intestinal paracellular permeability

Fifteen days after the start of the nutritional period, rats were housed in metabolic cages to collect and measure 24 h-urine excretion of an orally administered saline solution of 51-chromium-labelled ethylenediamine tetra-acetic acid (^51^Cr-EDTA, Perking Elmer Science). Radioactivity in urine was determined using a gamma counter (Cobra II; Packard) and expressed as the percentage of total radioactivity orally administered.

### H&E staining of colon

Segments of colonic tissue were excised, rinsed with phosphate-buffered saline, fixed in 4% paraformaldehyde and then embedded in paraffin. Serial slide-mounted tissue sections (5 μm thick) were deparaffinized in xylene, rehydrated through graded ethanol washes, and stained with hematoxylin and eosin (H&E) for histologic assessment.

### Intestinal mucosal expression profile of genes related to haem catabolism, aldehydes detoxification, inflammation, and barrier permeability

RNA from frozen mucosa was extracted with Tri reagent (Molecular Research Center), and 1 µg was reverse-transcribed with the iScript™ Reverse Transcription Supermix (Bio-Rad) for real-time quantitative polymerase chain reaction (qPCR) analyses. The primers for SYBR Green assays are listed in Table S3 as supporting information. Amplifications were performed using a ViiA 7 Real-Time PCR System (Applied Biosystems). The qPCR data were normalised to the level of RNA Polymerase II Subunit A (POLR2A) messenger RNA (mRNA) and analysed using LinRegPCR v.11 software.

### High-throughput 16S rRNA gene amplicon analysis

Genomic DNA from snap-frozen faecal samples was purified using columns (ZymoBIOMICS DNA miniprep Kit D4300, Zymo Research) after mechanical lysis. Hypervariable V3-V4 regions of the 16S rRNA gene were amplified using a two-step PCR as described previously^14^. Amplicons purity was checked using a TapeStation (4200, Agilent) before library preparation and sequencing (Illumina Miseq cartridge) was performed by the Genotoul facility (Get-PlaGe). Raw sequences were processed using the FROGS pipeline (Galaxy Version 3.2.3) as follows^59^: Each pair-end valid denoised sequences were filtered, merged and clustered with the swarm fastidious option using a maximum aggregation distance of 1^60^. Putative chimera were removed (vsearch) and clusters (i) whose abundance represented at least 0.005% of all sequences, (ii) present in at least three times in a minimum of 5% of total samples with a prevalence threshold of 5% of all samples, were retained, yielding to 486 final clusters. The Silva 138.1_16S reference database was used for cluster affiliation into Operational Taxonomic Units (OTUs) using BLAST+with equal multi-hits. Taxonomic multi-affiliations were manually checked. Once samples with valid sequences number lower than 15,000 were excluded (*n* = 6–8 per group), sample depth was normalised to 21,350 valid sequences. The corresponding 486 OTUs were agglomerated at the family and species ranks, reducing the taxon lists to 46 and 144, respectively (Table S4, Supporting information).

The R package Phyloseq (v1.34.0) was used to assess community profiles: Within-sample community richness and evenness (α diversity) were estimated using both the Chao-1 and Simpson indices, respectively, and examined by two-way ANOVA analysis and Holm-Šídák’s multiple comparisons test. Divergence of bacterial composition between samples (β diversity) was explored using Unifrac distance matrices, visualised using multidimensional scaling and statistically tested using permutational multivariate analysis of variance (Adonis test with 9999 permutations followed by pairwise multilevel comparison).

The difference in relative abundance of the taxonomic features was determined between a set of pairs of groups according to dietary calcium content or iron form by LEfSe^61^, with an alpha *p* value < 0.05 or 0.01 (Kruskal-Wallis sum-rank test), a *q* value < 0.05 (Wilcoxon rank-sum test) and a threshold on the logarithmic LDA score for discriminative features of 3.

To further explore how microbiota composition may reflect the physiological variables that results from diet contents (iron form and/or calcium source), covariances between bacterial taxa and measured physiological variables data sets were maximised using the R package MixOmics v6.15.45^62,63^. To this aim, raw 16S counts were transformed according to the mixMC preprocessing step prior to apply a PLS1 regression (Projection to Latent Structure)^64^. Outputs of data integration were visualised using a relevance Network graph, and corresponding relevant normalised bacterial taxa at the family or species level were shown.

### Statistical analyses

GraphPad Prism version 9.2 was used for most variables, except for microbiota and associated multivariate analyses. Results are expressed as mean ± s.e.m unless otherwise stated. n refers to the number of rats in each group. Two-way analysis of variance (ANOVA, 2 factors: iron, calcium and their interaction were tested) followed by Holm-Šídák’s multiple comparisons post-test was used to compare the experimental groups (haemoglobin *vs*. ferric citrate for each calcium source) and results were described as follow: *F*_*iron*_, *F*_*calcium*_ or *F*_*iron* × *calcium*_ and *p*_*iron*_, *p*_*calcium*_, *p*_*iron x calcium*_ referred to F-statistic and *p* values respectively resulting from ANOVA, whereas *p*_*adj*_ *(*_*Control, Mineral or Dairy Calcium*_*)* referred to *p* value resulting from post-tests. Two-sided analyses were used throughout, and *p* values less than or equal to 0.05 were considered significant. Detailed results of the statistical analyses are reported in Supporting Information Table S1.

### Reporting summary

Further information on research design is available in the Nature Research Reporting Summary linked to this article.

### Supplementary information

Reporting Summary

Supplementary Information

## Data Availability

Supporting information accompanies this paper at https://entrepot.recherche.data.gouv.fr (10.57745/XYPYPY). Paired raw sequences (16S rRNA sequences) have been submitted to ENA database under project accession code PRJEB71944. Further information and requests for resources and reagents will be fulfilled by the corresponding author.
