# Supplementary material for: Calcium-rich dairy matrix protects better than mineral calcium against colonic luminal haem-induced alterations in male rats
Source: NPJ Sci Food. 2024 Jul 2;8:43. doi: 10.1038/s41538-024-00273-y (PMC11220098; doi:10.1038/s41538-024-00273-y)
Supplement: Supplementary file 1 — Reporting Summary [file 41538_2024_273_MOESM1_ESM.pdf]

nature portfolio

Corresponding author(s): Fabrice PIERRE

Last updated by author(s): 2024 02 20

Reporting Summary

Nature Portfolio wishes to improve the reproducibility of the work that we publish. This form provides structure for consistency and transparency in reporting. For further information on Nature Portfolio policies, see our Editorial Policies and the Editorial Policy Checklist.

Statistics

For all statistical analyses, confirm that the following items are present in the figure legend, table legend, main text, or Methods section.

n/a	Confirmed	
☐	☒	The exact sample size (*n*) for each experimental group/condition, given as a discrete number and unit of measurement
☐	☒	A statement on whether measurements were taken from distinct samples or whether the same sample was measured repeatedly
☐	☒	The statistical test(s) used AND whether they are one- or two-sided<br>*Only common tests should be described solely by name; describe more complex techniques in the Methods section.*
☐	☒	A description of all covariates tested
☐	☒	A description of any assumptions or corrections, such as tests of normality and adjustment for multiple comparisons
☒	☐	A full description of the statistical parameters including central tendency (e.g. means) or other basic estimates (e.g. regression coefficient) AND variation (e.g. standard deviation) or associated estimates of uncertainty (e.g. confidence intervals)
☐	☒	For null hypothesis testing, the test statistic (e.g. *F*, *t*, *r*) with confidence intervals, effect sizes, degrees of freedom and *P* value noted<br>*Give P values as exact values whenever suitable.*
☒	☐	For Bayesian analysis, information on the choice of priors and Markov chain Monte Carlo settings
☒	☐	For hierarchical and complex designs, identification of the appropriate level for tests and full reporting of outcomes
☒	☐	Estimates of effect sizes (e.g. Cohen's *d*, Pearson's *r*), indicating how they were calculated

Our web collection on statistics for biologists contains articles on many of the points above.

Software and code

Policy information about availability of computer code

Data collection	*Provide a description of all commercial, open source and custom code used to collect the data in this study, specifying the version used OR state that no software was used.*
Data analysis	*Provide a description of all commercial, open source and custom code used to analyse the data in this study, specifying the version used OR state that no software was used.*

For manuscripts utilizing custom algorithms or software that are central to the research but not yet described in published literature, software must be made available to editors and reviewers. We strongly encourage code deposition in a community repository (e.g. GitHub). See the Nature Portfolio guidelines for submitting code & software for further information.

Data

Policy information about availability of data

All manuscripts must include a data availability statement. This statement should provide the following information, where applicable:

- Accession codes, unique identifiers, or web links for publicly available datasets
- A description of any restrictions on data availability
- For clinical datasets or third party data, please ensure that the statement adheres to our policy

Supporting information accompanies this paper at https://entrepot.recherche.data.gouv.fr (https://doi.org/10.57745/XYPYPY ; Reviewer access: https://entrepot.recherche.data.gouv.fr/privateurl.xhtml?token=69cf99e8-3ce6-43d2-9fe7-8983121f25ae). Paired raw sequences (16S rRNA sequences) have been

submitted to ENA database under project accession code PRJEB71944 . Further information and requests for resources and reagents will be fulfilled by the corresponding author.

Research involving human participants, their data, or biological material

Policy information about studies with human participants or human data. See also policy information about sex, gender (identity/presentation), and sexual orientation and race, ethnicity and racism.

Reporting on sex and gender	*Use the terms sex (biological attribute) and gender (shaped by social and cultural circumstances) carefully in order to avoid confusing both terms. Indicate if findings apply to only one sex or gender; describe whether sex and gender were considered in study design; whether sex and/or gender was determined based on self-reporting or assigned and methods used. Provide in the source data disaggregated sex and gender data, where this information has been collected, and if consent has been obtained for sharing of individual-level data; provide overall numbers in this Reporting Summary. Please state if this information has not been collected. Report sex- and gender-based analyses where performed, justify reasons for lack of sex- and gender-based analysis.*
Reporting on race, ethnicity, or other socially relevant groupings	*Please specify the socially constructed or socially relevant categorization variable(s) used in your manuscript and explain why they were used. Please note that such variables should not be used as proxies for other socially constructed/relevant variables (for example, race or ethnicity should not be used as a proxy for socioeconomic status). Provide clear definitions of the relevant terms used, how they were provided (by the participants/respondents, the researchers, or third parties), and the method(s) used to classify people into the different categories (e.g. self-report, census or administrative data, social media data, etc.) Please provide details about how you controlled for confounding variables in your analyses.*
Population characteristics	*Describe the covariate-relevant population characteristics of the human research participants (e.g. age, genotypic information, past and current diagnosis and treatment categories). If you filled out the behavioural & social sciences study design questions and have nothing to add here, write "See above."*
Recruitment	*Describe how participants were recruited. Outline any potential self-selection bias or other biases that may be present and how these are likely to impact results.*
Ethics oversight	*Identify the organization(s) that approved the study protocol.*

Note that full information on the approval of the study protocol must also be provided in the manuscript.

Field-specific reporting

Please select the one below that is the best fit for your research. If you are not sure, read the appropriate sections before making your selection.

☒ Life sciences ☐ Behavioural & social sciences ☐ Ecological, evolutionary & environmental sciences

For a reference copy of the document with all sections, see nature.com/documents/nr-reporting-summary-flat.pdf

Life sciences study design

All studies must disclose on these points even when the disclosure is negative.

Sample size	For an experimentation of nutrition in rats, the number of rats is defined to evidence differences in fecal, urinary biomarkers and microbiota composition. From a statistical point of view, a power test was carried out using previous data (our own or published) to calculate the number of animals required. For the consequences on faecal and urinary markers, the monitoring of TBARs, DHN-MA justifies a number of 5 rats / experimental group. But, for data on permeability, the in vivo variability of this parameter measured with EDTA 51Cr is greater and justifies the use of 8 rats/group. In the same way, for data on microbiota, the in vivo variability of this parameter measured by amplification of bacterial 16S RNA justifies the use of 8 rats/group. For this study, 8 rats per group were therefore chosen.
Data exclusions	The exclusion criteria are linked to the quality checks specific to the analysis softwares and pipelines used (TECAN, LingReg, FROGS, MixOmics, etc.)
Replication	For this animal study, the unit of analysis was the individual rats:<br>-for microbiota, DHN-MA and for permeability, n=8<br>-for fecal water samples; n=8 and each sample was measured in triplicate after three individual preparation
Randomization	At reception, rats were placed randomly, one by one, in each cage. When each cage contained one rat, a second rat was placed in it, sequentially. After acclimation to the animal colony, the experimental groups were decided at random, by placing labels on the cage, in a "random" order, avoiding to place two label of same group on next cages, or in the same "raw" or the same column of cages. Rats were then weighted, and some changes were made so that no statistical difference could be seen in mean body weights at the beginning of the study.
Blinding	Each sample (feces, urine, colon) was given a two-letter code randomly generated by a computer. Codes were kept in a file that was known only to senior investigators, who did not participate to laboratory analyses. Code was broken only before statistical testing.<br>All analytical techniques (biochemical assay of fecal water and urine) were done by a unique investigator, blinded for the origin of the sample.

Reporting for specific materials, systems and methods

We require information from authors about some types of materials, experimental systems and methods used in many studies. Here, indicate whether each material, system or method listed is relevant to your study. If you are not sure if a list item applies to your research, read the appropriate section before selecting a response.

Materials & experimental systems

n/a	Involved in the study
☒	☐ Antibodies
☒	☐ Eukaryotic cell lines
☒	☐ Palaeontology and archaeology
☐	☒ Animals and other organisms
☒	☐ Clinical data
☒	☐ Dual use research of concern
☒	☐ Plants

Methods

n/a	Involved in the study
☒	☐ ChIP-seq
☒	☐ Flow cytometry
☒	☐ MRI-based neuroimaging

Animals and other research organisms

Policy information about studies involving animals; ARRIVE guidelines recommended for reporting animal research, and Sex and Gender in Research

Laboratory animals	Male Fischer 344 (F344/DuCrl) rats were purchased from Charles River Laboratories, 8 rats/group, aged 6 weeks.
Wild animals	The study did not include wild animals
Reporting on sex	Only male F344 rats were integrated in this study.<br>The menstrual cycle has a strong impact on the response to pro-inflammatory stimuli and the antioxidant response, so we decided to work with male rats to avoid this additional bias in the interpretation of our results.
Field-collected samples	The study did not involve field collected samples
Ethics oversight	The experimental protocol was approved by the Animal Care Use Committee (Comité d'Ethique Pharmacologie-Toxicologie-Occitanie Toulouse, registered as no.86 at the Ministry of Research) under the authorisation number [#16138-TOXCOM 214FP], and conducted in accordance with the European Union guidelines.

Note that full information on the approval of the study protocol must also be provided in the manuscript.
