# Supplementary material for: Calcium-rich dairy matrix protects better than mineral calcium against colonic luminal haem-induced alterations in male rats
Source: NPJ Sci Food. 2024 Jul 2;8:43. doi: 10.1038/s41538-024-00273-y (PMC11220098; doi:10.1038/s41538-024-00273-y)
Supplement: Supplementary file 2 — Supplementary Information [file 41538_2024_273_MOESM2_ESM.pdf]

Supporting information and raw data accompany this paper at

https://entrepot.recherche.data.gouv.fr (https://doi.org/10.57745/XYPYPY).

Supplementary Tables

Table S1: F and p values resulting from 2-way ANOVA analysis and Holm-Šídák's multiple comparison tests

**Figure**	**F value** Iron x calcium	***p* value** F iron x calcium	**F value** F calcium	***p* value** F calcium	**F value** F Iron	***p* value** **F iron**	**Post-test comparison Ferric citrate vs Haemoglobin**	***p adj***
**Figure 1**								
1a	0,5	0,6038	0,95	0,954	0,9	0,8665	Control	0,8578
Body weight							Mineral Calcium	0,8578
							Dairy Calcium	0,8578
1b	2,9	0,0709	12,2	**<0,0001**	36,9	**<0,0001**	Control	**<0,0001**
Heme							Mineral Calcium	**0,0081**
							Dairy Calcium	**0,0502**
1c	21,3	**<0,0001**	32,6	**<0,0001**	16,2	**0,0003**	Control	**<0,0001**
Hmox1							Mineral Calcium	0,7537
							Dairy Calcium	0,9887
1d	4,8	**0,0138**	7,5	**0,0017**	34,7	**<0,0001**	Control	**<0,0001**
TBARS							Mineral Calcium	**0,0022**
							Dairy Calcium	0,2436
1e	2,9	**0,0685**	2,4	0,1035	20,1	**<0,0001**	Control	**0,0007**
DHN-MA							Mineral Calcium	**0,0071**
							Dairy Calcium	0,4992
**Figure 2**								
2a	0,35	0,7045	3,4	**0,0444**	0,08	0,7756	Control	0,8783
^{51}Cr							Mineral Calcium	0,8783
							Dairy Calcium	0,8783
2b	1,25	0,2994	5,2	**0,0107**	1,45	0,2364	Control	0,3368
IL-18							Mineral Calcium	0,562
							Dairy Calcium	0,3929
2c	2,4	0,1031	9,3	**0,0006**	2	0,1621	Control	0,3444
Slc7a11							Mineral Calcium	0,1694
							Dairy Calcium	0,2417
2d	0,9	0,4276	5	**0,0137**	1,9	0,1805	Control	0,2664
AKr1b8							Mineral Calcium	0,9283
							Dairy Calcium	0,9081
**Figure 3**								
3a	0,25	0,7796	4,5	**0,0184**	0,5	0,4842	Control	0,8648
Chao-1							Mineral Calcium	0,8648
							Dairy Calcium	0,8652
3b	3,8	**0,0312**	11	**0,0002**	0,5	0,4696	Control	0,2122
Simpson							Mineral Calcium	0,4768
							Dairy Calcium	0,0836
3c	1,3	0,0921	7,3	**<0,0001**	2	**0,0168**	Control	**0,01**
Unifrac, pairwiseAdonis							Mineral Calcium	0,071
							Dairy Calcium	0,178
**Figure 4**								
4b	8	**0,0014**	133,1	**<0,0001**	8,3	**0,0065**	Control	**0,0001**
Bacteroidota							Mineral Calcium	0,3926
							Dairy Calcium	0,3926
4c	6	**0,0059**	76	**<0,0001**	**0,02**	0,9	Control	**0,0229**
Firmicutes							Mineral Calcium	0,3231
							Dairy Calcium	0,1823
4d	2	0,1469	6,6	**0,0036**	16,3	**0,0003**	Control	**0,0416**
Actinobacteriota							Mineral Calcium	**0,0011**
							Dairy Calcium	0,4117
Proteobacteria	0,4	0,689	2,1	0,1385	6,5	**0,015**	Control	0,3352
							Mineral Calcium	0,3406
							Dairy Calcium	0,129
Deferribacterota	0,3	0,7191	1,9	0,1617	0,2	0,6923	Control	0,7804
							Mineral Calcium	0,9698
							Dairy Calcium	0,9927
Desulfobacterota	0,15	0,8625	17,8	**<0,0001**	1,3	0,295	Control	0,7352
							Mineral Calcium	0,6923
							Dairy Calcium	0,8592
**Figure 5**								
5b	1,3	0,274	8,5	**0,0009**	7,7	**0,0086**	Control	**0,0416**
Bifidobacteriaceae							Mineral Calcium	0,1402
							Dairy Calcium	0,7265
5c	0,7	0,5022	8,4	**0,001**	13,75	**0,0007**	Control	**0,0153**
Eubacterium coprostanoligenes							Mineral Calcium	0,1109
							Dairy Calcium	0,1587
5d	2,75	0,0773	46,1	**<0,0001**	7,9	**0,0079**	Control	**0,0044**
Muribaculaceae							Mineral Calcium	0,7149
							Dairy Calcium	0,7149
5e	10,8	**0,0002**	32,5	**<0,0001**	**0,001**	0,9689	Control	**0,049**
Peptococcaceae							Mineral Calcium	0,1933
							Dairy Calcium	**0,0017**
**Figure 6**								
6b	6,5	**0,004**	12,8	**<0,0001**	3	0,0905	Control	**0,0015**
Eisenbergiella							Mineral Calcium	0,7175
							Dairy Calcium	0,7959
Eubacterium coprostanoligene	0,7	0,5022	8,4	**0,001**	13,75	**0,0007**	Control	**0,0153**
							Mineral Calcium	0,1109
							Dairy Calcium	0,1587
Unknown Enterorhabdus	3,05	**0,0598**	5,3	**0,0094**	3,6	**0,0652**	Control	**0,0323**
							Mineral Calcium	0,4752
							Dairy Calcium	0,4143
Peptococcus spp.	2,4	0,1059	32,9	**<0,0001**	**0,015**	0,8976	Control	0,2954
							Mineral Calcium	0,5392
							Dairy Calcium	0,3471
Oscillobacter spp.	2,8	0,0734	1,9	0,1579	2,6	0,115	Control	**0,0243**
							Mineral Calcium	0,9951
							Dairy Calcium	0,9951
Multi-*Oscillospirales*	3,2	**0,0511**	0,5	0,5985	6,1	**0,0187**	Control	**0,0123**
							Mineral Calcium	0,6715
							Dairy Calcium	0,2632
Multi-*Lachnospiraceae*	3,2	**0,0514**	9,3	**0,0006**	6,8	**0,0134**	Control	**0,0047**
							Mineral Calcium	0,9487
							Dairy Calcium	0,608
6c	1	0,3838	7,6	**0,0017**	8,5	**0,0061**	Control	**0,0278**
Bifidobacterium pseudolongum							Mineral Calcium	0,4291
							Dairy Calcium	0,4291
Lachnoclostridium	0,6	0,5645	1,8	0,1732	7,3	**0,0106**	Control	**0,0738**
							Mineral Calcium	0,3573
							Dairy Calcium	0,3573
unk *Erysipelatoclostridiaceae*	1,7	0,2016	14,8	**<0,0001**	3,5	**0,0697**	Control	**0,0498**
							Mineral Calcium	0,9169
							Dairy Calcium	0,9169
Bacillus mycoides	1	0,3744	2,4	0,1018	3,7	**0,0619**	Control	0,3105
							Mineral Calcium	0,9969
							Dairy Calcium	0,1884
Anaerofustis spp.	0,6	0,5504	1,7	0,1997	6,9	**0,0124**	Control	0,0761
							Mineral Calcium	0,3507
							Dairy Calcium	0,3865
6d	10	**0,0004**	19,9	**<0,0001**	0,08	0,7889	Control	**0,056**
unk *Peptococcaceae*							Mineral Calcium	0,1111
							Dairy Calcium	**0,0039**
Lactobacillus intestinalis	4,8	**0,0145**	7,6	**0,0017**	38,8	**<0,0001**	Control	**<0,0001**
							Mineral Calcium	**0,0371**
							Dairy Calcium	**0,0296**
Eubacterium brachy	2,2	0,1268	15,3	**<0,0001**	10,1	**0,003**	Control	**0,0051**
							Mineral Calcium	0,2895
							Dairy Calcium	0,5514

**Sup. Figure**	**F value** Iron x calcium	***p* value** F iron x calcium	**F value** F calcium	***p* value** F calcium	**F value** Iron	F ***p* value** F iron	**Post-test comparison Ferric citrate vs Haemoglobin**	***p* adj**
**Sup Figure (heme impact not normalized by calcium)**								
a	0,5	0,5966	4,7	**0,0155**	22,8	**<0,0001**	Control	**0,0384**
Butyricoccus UCG-009							Mineral Calcium	**0,0306**
							Dairy Calcium	**0,0029**
b	0,5	0,6107	29,6	**<0,0001**	26,7	**<0,0001**	Control	**0,0049**
Coriobacteriaceae UCG-002							Mineral Calcium	**0,0049**
							Dairy Calcium	**0,0357**
c	4,1	**0,0241**	15,6	**<0,0001**	0,3	0,5761	Control	**0,0465**
Candidatus Soleaferrea massiliensis							Mineral Calcium	0,799
							Dairy Calcium	0,3068
unknown *Lactobacillus*	1,2	0,3037	5,5	**0,0079**	11,9	**0,0014**	Control	**0,0457**
							Mineral Calcium	**0,0195**
							Dairy Calcium	0,4637
**Sup Figure (calcium impact without haem alteration)**								
a	0,8	0,4639	30,6	**<0,0001**	**0,01**	0,9076	Control	0,6534
Desulfovibrio spp.							Mineral Calcium	0,8056
							Dairy Calcium	0,8056
b	0,5	0,6036	21,2	**<0,0001**	7,4	**0,01**	Control	0,0814
Multi-*Enterococcus*							Mineral Calcium	0,332
							Dairy Calcium	0,332
Negativibacillus spp.	0,7	0,4803	38,5	**<0,0001**	0,1	0,7444	Control	0,8063
							Mineral Calcium	0,8063
							Dairy Calcium	0,8063
c	0,1	0,8981	28,8	**<0,0001**	0,4	0,5065	Control	0,9676
Frisingicoccus caecimuris							Mineral Calcium	0,8224
							Dairy Calcium	0,9676
Macrococcus spp.	0,23	0,7932	50,9	**<0,0001**	0,23	0,633	Control	>0,9999
							Mineral Calcium	>0,9999
							Dairy Calcium	0,7926
Exiguobacterium spp.	1,1	0,3441	61,7	**<0,0001**	1,1	0,3027	Control	>0,9999
							Mineral Calcium	>0,9999
							Dairy Calcium	0,2149

Table S2: mucosal gene expression related to inflammation and detoxification pathways

Rat	Calcium	Iron	Akr1b1	Akr1b7	Akr1b8	Akr1b10	Aldh2	Aldh3A2	Hmox1	xCT/Slc7a11	Keap1	Nqo1	Nrf2	Cox2a	Ptgr1	Gclc	Gsr	Gclm	Gsta4	Ppar-g	Cldn1	Occl	ZO1	Tnf	Ing	Il6	Il18	Il15	Il10	Il12
1	Control	Control	0,7144929	2,43978708	3,1693303	1,39814427	5,71412477	1,46859249	0,25050082	0,066475259	0,55401724	0,58160807	4,20442358	0,39875925	29,1745066	1,27150892	1,59504507	6,97531407	1,14117918	11,7478913	0,02252767	34,7652267	1,85608183	0,14861139	0,01722076	0,01106794	4,71923535	2,67126529	0,05787741	0,05048691
2	Control	Control		0,97363388		0,51168215	3,86229234	0,6467721	0,13804768	0,045825725	0,29478023	0,25360296	1,93599327	0,23069809	14,0272176	0,67763001	1,14652531	2,67450842	0,91857399	6,33174303	0,02653001	21,0608157	1,30748474	0,18663552	0,0120002	0,02051448	3,18514331	1,86635516	0,09686538	0,05275153
3	Control	Control	0,62640117	1,81259599	2,33026743	1,30824488	7,72572884	0,99395356	0,37867063	0,042588688	0,78406212	1,09229141	3,75945421	0,30611393	25,984976	1,82192389	1,3190685	3,97720595	0,74670296	10,1114366	0,01018186	48,2727681	2,13312005	0,12882736	0,00878856	0,00499662	1,51334635	0,90672168	0,00938875	0,01796295
4	Control	Control	0,73289667			1,27295873	7,39241697	1,00592757	0,44929303	0,079041606	0,52538378	1,15338406	4,28439424		19,9186075	1,41915368	1,13662744	6,62195768	1,04895457	16,2190131	0,01010894	37,7069529	1,85703288	0,0938807	0,00234366	0,00526402	0,76948642	0,5683135	0,00883216	0,00757751
5	Control	Control	0,36718147	1,29243814	1,66664159	0,9045605	3,99588985	0,7829786	0,42194653	0,07289342	0,42668649	0,3867935	2,49093454	0,236783	14,9511626	0,9076674		4,59767117	1,10274901	5,82223679	0,01806038	30,6525887	1,27679452	0,23600815	0,01842015	0,01629751	4,01501678	2,12982969	0,06223996	0,06036916
6	Control	Control	0,17849572	0,46481816	0,49225889	0,25884845	2,48730663	0,3138246	0,15963761	0,036344584	0,10023853	0,23503079	1,04736744	0,10734025	4,40966205	0,41562668	0,4183372	1,33489544	0,71098681	6,13529652	0,02860146	19,3089501	1,20071894	0,15889702	0,01076969	0,01288831	0,57556031	1,25352093	0,03834215	0,01253521
7	Control	Control	0,56019028	1,87295811	2,68328834		5,81360758		0,24457253	0,04743704	0,45666196	0,90732621		0,56899404	25,2567812	1,04013159	1,19042504	3,96616373	0,85395639	12,2314674	0,00968133	40,4010094	2,59472937	0,13792574	0,00911769	0,00794731	1,72407171	1,47540752	0,01312947	0,02105357
8	Control	Control												0,16551471	9,77624577					7,14102086	0,00813823	22,7852577	1,5435288	0,17333754		0,01175962	1,12561511	1,57514192	0,04491043	0,0127306
1	Control	Hemoglobin	0,36429802	2,34836893	3,13986414	1,28121279	5,10072247	1,0895418	0,69635262	0,05967958	0,36158171	0,31483475	2,20536434	0,27065271	25,3483994	0,70103547	1,35165602	4,63234728	0,74179108	8,50391777		28,2252046	1,51247065							
2	Control	Hemoglobin	0,26520947	1,34938576		0,7752422	4,20485456	0,65629313	0,66974365		0,31865183	0,33169784	1,77697994			0,63811478	0,9543764	3,02400614	0,637676	7,18597353	0,00407449	17,7138223	1,37127507	0,03066818	0,00577	0,00355751	1,14786608	1,06024272	0,00670177	0,00588829
3	Control	Hemoglobin	0,36231309	1,86850275		0,97364273	4,51207208		0,67905063	0,082728743	0,36769317	0,34735534	2,38211946	0,12658459	20,0528778	0,79605721	1,19033609	4,4973857	0,93878195	8,52655557	0,02000671	31,5661351	1,52250266	0,19237225	0,01888746	0,01860764	3,69354712	2,18255057	0,07925737	0,02532318
4	Control	Hemoglobin	0,22787093	1,55814361	2,03187011	0,77533772	3,97128113	0,63947956	0,64474053	0,066992741	0,26911083	0,34147008	1,85078643	0,0790853	16,0912589	0,64144988	0,77315984	4,93680185	0,93970901	6,97787587		23,3312681								
5	Control	Hemoglobin												0,14052839	21,1099737					8,84376663	0,01516148	27,1917103	1,74033907	0,2409064		0,01519437	5,98566062		0,05443005	0,03806814
6	Control	Hemoglobin	0,37067005	2,14516557	2,80357797	1,17997127	5,06741757	0,68044437	1,10602469	0,047158073	0,38726455	0,88849677	2,43352405	0,22702574	19,5231458	1,11571886	1,13324336	5,2527798	1,0240807	10,3312851	0,02532623	25,5366605	1,43315379	0,10810349	0,00518162	0,00970788	1,54346062	0,78581745	0,0368793	0,01013662
7	Control	Hemoglobin	0,54629332	1,90360667	3,53875458	1,50953535	4,98307217	0,71643417	1,03997321	0,060241849	0,4593791	0,88880462	2,13150141	0,21220637	17,3650773	1,1616152		5,94209857	1,27989297	6,52773872	0,01602734	25,0059472	1,39298238	0,0777834	0,01243544	0,00671315	2,16505397	0,99830292	0,03071701	0,0296766
8	Control	Hemoglobin	0,45419516	2,21681887		1,55900372	5,9374054	1,43426681	0,63894099	0,067770686	0,54492591	0,60403051	2,77514824	0,26035015	21,3975738	1,18155795		6,19958895	1,46113829	7,76497505	0,01138831	31,8171341	1,54143339	0,08776267	0,00919806	0,01427986	4,93950863	2,60188109	0,04141666	0,03374315
1	Mineral Calcium	Control	0,43852393		1,93079846	0,94410995	4,16146489	0,61539886	0,21745401	0,02707499	0,27107584	0,22078748	2,16718617		12,9849664	0,82928396	0,97254691	4,14702734	0,96679383	10,2682087	0,00680364	29,688645	1,49792846	0,24435999	0,01619915	0,0199881	1,63089702	1,24101928	0,03151969	0,03492426
2	Mineral Calcium	Control	0,2562084	1,18303352	1,9542299	0,82679978	4,17785312	0,56729678	0,23509356	0,063240349	0,25481902	0,27765937	1,37384463	0,16680426	7,67636739	1,04873656	1,05038776	2,2568699	1,42281053	5,57832932	0,01992856	15,2957996	0,96990252	0,48131677	0,13855779	0,01970327		2,42706544	0,11592566	0,04864372
3	Mineral Calcium	Control	0,54633667	2,22754386	2,99657862	1,27569345	10,5684515	0,74827655	0,35819479	0,063104294	0,55508263	0,7377295	2,85761572	0,35610139	20,5908319	1,4366004	1,55569625	2,92741631	0,84232922	14,248768	0,03478725	44,5524232		0,13743415	0,00698195	0,00335624		0,62715051	0,02737268	0,00546307
4	Mineral Calcium	Control	0,50646072	1,27893049	1,60986368	0,75268981	4,83068694	0,71209925	0,3013409	0,062779013	0,34486097	0,31472344	2,98663505	0,20483527	13,8349768	1,18191658	1,23507315	4,86480389	1,77192109	10,4057647	0,03146978	37,7281289	1,88924054	0,21659361	0,01639316	0,01257092	1,59684698	1,84741606	0,04603676	0,04714096
5	Mineral Calcium	Control	0,32012701	0,71352344	1,11488609		4,5790069	0,56493232	0,25118479	0,039041434	0,44861135	0,32273839		0,1322136	8,17319246	1,42476239		3,16569101	1,32279641	7,86495045	0,0218419	49,6586126	1,28045549	0,12049503	0,00934097	0,0052747	1,13550074	1,31954215	0,03614091	0,01382047
6	Mineral Calcium	Control	0,28734981	1,42545014		0,83990057	4,34570315	0,53522101	0,12762811	0,024462752	0,3171279	0,26518501	2,25056219	0,20501903	8,90011415	0,62003569	0,9481428	2,59459301	0,9989099	7,60784961	0,01387243	22,7545925	1,20965081	0,18472113	0,00999451	0,01326689	1,40681659	2,16527422	0,01456973	0,03413059
7	Mineral Calcium	Control	0,40971005		1,23643169	0,53509276	4,35384119	0,4536005	0,30011997	0,030672038	0,30436927	0,57867793	2,28913744	0,27734869	12,2806147	1,24072798		3,31001664	1,09188123	13,9938828	0,00896556	47,2803074	2,526934	0,10135284	0,02207548	0,00905928	1,88474632	2,34638769	0,01655661	0,02894804
8	Mineral Calcium	Control																												
1	Mineral Calcium	Hemoglobin	0,52911055	1,90570384	2,20641542	1,02236895	3,71082644	0,75779101	0,29319617		0,37832099	0,47143277	2,58601615	0,34156041	8,30036003	1,11475969	1,37123698	3,130689	1,65609634	9,56577259	0,01013567	25,4130659	1,60638841	0,14297658	0,01508109	0,00845618	1,12618543	1,54728085	0,01870447	0,03780066
2	Mineral Calcium	Hemoglobin	0,20752573	0,98507742	1,41092491	0,56966187	3,91200921	0,39425381	0,30655055	0,024860212	0,24226153	0,29993265	1,86293382	0,0739278	8,81035973	0,51498529	0,69017594	1,59771983	0,7134451	4,88387486	0,02379657	18,8898229	1,20937853	0,1672647	0,02052155	0,01069651	0,99866868	1,39785503	0,03577216	0,03549104
3	Mineral Calcium	Hemoglobin	0,38224489	1,30539261	1,7467233	0,71679868	6,95795193	0,47961352	0,25245299	0,012755472	0,48338929	0,59393949	2,33076502	0,1694732	12,4547144	1,00245424	1,1062647	2,79360641	1,14580114	9,90195885	0,00999761	26,7553353	2,0648171	0,07465581	0,0151922	0,00679211	0,91623041	1,1276682		0,00866634
4	Mineral Calcium	Hemoglobin	0,24029287	0,82365218	1,19436762	0,46085336	4,1756123	0,48520493	0,11513242	0,032389537	0,25336059	0,25393636	1,61344737		7,41330293	0,52798998	0,73511341	2,1727779	1,20904637	5,87154184	0,05382059	16,4559719	1,19853982	0,37680415	0,16260179	0,02850031	1,66201831		0,15660173	0,06300069
5	Mineral Calcium	Hemoglobin	0,28721426	1,14127205	1,67003249	0,67119222	4,02735308	0,44735442	0,237383	0,030147362	0,35472731	0,37018838	2,02750383		11,8632552	0,77690239	0,8207277	2,60047311	1,12426791	8,60901625	0,01430346	24,8986584	1,45191207	0,15736918	0,04269223	0,00947331	0,27360424	1,10314237	0,00774483	0,00464629
6	Mineral Calcium	Hemoglobin	0,36755534	2,5374872	2,89832453	1,34454167	5,00882882		0,21284558	0,050204177	0,47530908	0,84919695	3,0697655	0,23221096	14,6092768	0,95252748	1,03881891	4,62341394	0,94687548	10,496589	0,01724745	30,0449318		0,12138968	0,01218955	0,01164835	2,51377802	2,50872012		0,03267406
7	Mineral Calcium	Hemoglobin	0,29763395		1,10206829	0,67243445	6,04809967	0,62259111	0,08470358	0,01964362	0,36568248	0,34973499	2,45967002	0,24185888	6,09395347	0,76155115	0,57351244	2,23151107	0,79634626	6,8948849	0,01264103	22,389074	1,50652333	0,13773067	0,00323573		0,2646127		0,00637627	0,00330176
8	Mineral Calcium	Hemoglobin	0,86663392	2,04429576	2,49740812	1,10218258	4,65588198	0,61119799	0,20597521	0,0269969	0,18779628	0,47198385	3,72111074	0,3837654	18,0190685	1,06037728	1,76437641	5,91184412	3,89241661	18,964948	0,0183149	48,2405141	1,87290777	0,18115104	0,03611366			5,01162334	0,02424436	0,03208438
1	Dairy Calcium	Control												0,16266173	13,1239653					10,5808828	0,01611168	34,2075963		0,07095996	0,01467191	0,00323605	0,18579854	0,40107804	0,01302131	0,00409305
2	Dairy Calcium	Control	0,27602834	0,93903925	1,25899142	0,55173626	3,71221666	0,45137201	0,17724988	0,029510524	0,31650088	0,11875644	1,63896267	0,26310852	9,44114727	0,6659676	0,72686958	2,10075266	0,74235295	7,53232749	0,0104614	22,791683	1,47503955			0,01854819	1,77821919	2,31989212	0,03151552	0,03501724
3	Dairy Calcium	Control	0,43763026	0,96359769	1,21084909	0,51789541	4,88812557	0,77464495	0,27567375	0,038223146	0,38715775	0,45618078	2,44152176	0,17096793	9,93051873	0,8923484	1,18113659	3,15116306	0,70743783	12,1777194	0,00816584	37,4859657		0,33051072	0,08941236	0,0068816	0,50642772	0,95954725	0,09290424	0,00879585
4	Dairy Calcium	Control	0,63507188	1,5640348	1,82444779	0,82917548	6,85303754	0,60797494	0,18333391	0,070445327	0,40579195	0,51792952	2,82538594	0,37104835	10,7969765	1,11069459	1,21546101	2,72469806	0,85709523	10,2174467	0,02678093	34,7744889	1,96122575	0,07747009	0,00254836	0,00543959	0,23352229	0,47353131	0,01679441	0,01072164
5	Dairy Calcium	Control	0,26883816	0,48330285	0,61201813	0,24388572	3,5188385	0,5483793	0,20103607	0,020644029	0,21680421	0,15659177	1,74936032	0,24959528	6,07691828	0,98629372	0,60174498	2,42144851	1,04425719	7,69199243		40,2360278	1,31144142			0,01071278	2,10732575	2,20518917	0,03281687	
6	Dairy Calcium	Control												0,5484579	12,4103089					8,9507315	0,05751548	29,2698471		0,16022708	0,02142659	0,02350501	2,03307004	2,17666979	0,07013714	0,12614104
7	Dairy Calcium	Control	0,39407632	1,09702891	1,5971745	0,65881711	3,9551932	0,62364723	0,17673872	0,058039808	0,33505892	0,32634521	2,11304299	0,19117606	11,7980847	0,72057549		2,80655869	1,08223259	9,40411996	0,01964558	27,9979094	1,48781811	0,06655745	0,00407015	0,01036713	0,55423302	1,23217877	0,04175702	0,01783938
8	Dairy Calcium	Control	0,50729921	1,46764093	1,97018871	0,69876718	5,25973015		0,21510825	0,053516607	0,67593049	0,36404733	3,32304565	0,29389355	20,054926	0,96255512		4,27472953	0,79884952	11,5358795	0,00692892	23,8246661	1,88649386	0,07151632	0,00690417	0,00456071	1,23483202	0,89086078	0,01621353	0,00368717
1	Dairy Calcium	Hemoglobin	0,52008051	1,92719697		1,21431281	5,66385569	0,65322276	0,14175455	0,024310222	0,23244867	0,60200858	3,23443149	0,18938544	14,2868455	0,90936751	1,05790127	4,73105301	1,14645266	15,3028532	0,00367087	31,8504221			0,00627087	0,00981632	1,06363548	1,1046373	0,01372245	0,00423042
2	Dairy Calcium	Hemoglobin	0,28387671	1,9761063	2,35821132	1,17670682	5,1665323	0,6027656	0,12258787	0,011499596	0,27954114	0,49230783	2,60508209	0,20921172	10,9195745	0,71387137	1,00018442	3,64990582	0,88715397	9,21035582	0,01023874	26,7701584	1,59183395	0,04163343	0,01602887	0,01531185	2,15106059	2,08938143	0,04086244	0,03161057
3	Dairy Calcium	Hemoglobin	0,35046086	1,70649929	1,98364026	0,98025017	4,56963214	0,64427254	0,31711934	0,041478396	0,31277423	0,45422864		0,11416005		0,85342024	0,96186538	3,49565621	0,75611358	14,1325826	0,00913468	33,3614943		0,16339502	0,01891265	0,01395934	3,33866195	4,44511639	0,02939824	0,03345095
4	Dairy Calcium	Hemoglobin	0,49632016	1,35792835	1,6036928	0,77377927	7,40031889	0,52740985	0,45130688	0,037069781	0,5395427	1,52607374	2,99222342	0,24738515	12,206523	1,44552991	1,25936257	3,04114493	1,07950444	12,0229624	0,01023874	36,1495682	1,78991691	0,04163343	0,01602887	0,01531185	2,15106059	2,08938143	0,04086244	0,03161057
5	Dairy Calcium	Hemoglobin		1,39601254		0,65290986	4,80029552	0,78247214	0,25261895	0,040962299	0,49974458	0,67595945	3,70120957		9,37884022	1,37692001		4,43700455	1,67984238	9,00982453		26,3464614	1,37613553	0,09749082	0,01123682	0,00875142	0,74562102	1,05192073	0,02021214	
6	Dairy Calcium	Hemoglobin		0,54794527	0,79480015	0,35424936		0,35636984	0,08038452	0,04946672	0,20814794	0,25161325	1,38537242	0,1508009	5,80192486	0,40252158	0,53590448	1,35853194	0,97070782	4,33834397	0,08398498	16,5854896	0,85480399	0,18908483	0,00915768	0,04163662	3,34516445	2,76391247	0,06358185	0,10616745
7	Dairy Calcium	Hemoglobin	0,28799724	0,5016531	0,63882801	0,37865715	4,70492572	0,40702835	0,07180485	0,041557604	0,40857406	0,34144013	1,73632525	0,12973601	5,1661229	0,62957207	0,68389457	0,96595507	0,92287511	5,43072672	0,00828261	18,5693442		0,02707549	0,00376509	0,006366	0,34481259	0,60727303	0,0071433	0,00417982
8	Dairy Calcium	Hemoglobin	0,44641794	1,32957631	2,05871804	0,67077811	5,02883825	0,69876227	0,19327339	0,009086489	0,3288529	0,38045216	2,7990664	0,13400114	13,0670291	1,04017784		4,20285648	1,59177601	11,0160071	0,00800289	33,4953535	1,56145716	0,11211356	0,01561112	0,01633467		3,74929964	0,04333986	0,03522734

Table S3: List of genes and primers sequences used in this study

Gene Name	Alias	Forward	Reverse
Akr1b1	**Aldo-Keto Reductase Family 1 Member B1**	TTCAAGCCTGGGCCTGACTA	AATCACGTTTCCCGATGCAT
Akr1b10	**Aldo-Keto Reductase Family 1 Member B10**	CCTGGGCACCTGGAAGACT	CATCAATGGCTGCCTTCACA
Akr1b7	**Aldo-Keto Reductase Family 1 Member B7**	CTTATTCAGGAGCCTTTCGATCTG	GGGCTGGTGAAAGCTTTGG
Akr1b8	**Aldo-Keto Reductase Family 1 Member B8**	GCCGCCAAGCACAAGAAAA	CCTCTGGATGTGGAACCGAAT
Aldh2	**Aldehyde Dehydrogenase 2 Family Member**	GCCGCAGACCGTGGTTACT	CGATGGTCATGCCATCTTTG
Aldh3A2	**Aldehyde Dehydrogenase 3 Family Member A2**	TCTGAGGCAGCGGTTTGATC	CCAACTGCGGTGTTTCCTGT
Hmox1	**Haem Oxygenase 1**	CAACCCCACCAAGTTCAAACA	AGGCGGTCTTAGCCTCTTCTG
xCT/Slc7a11	**Solute Carrier Family 7 Member 11 (xCT)**	CCTGGCATTTGGACGCTACAT	TCAGAATTGCTGTGAGCTTGCA
Keap1	**Kelch Like ECH Associated Protein 1**	CTGCATCCACCACAGCAGCGT	GTGCAGCACACAGACCCCGGC
Nqo1	**NAD(P)H Quinone Dehydrogenase 1**	CGCAGAGAGGACATCATTCA	GTGGTGATGGAAAGCAAGGT
Nrf2/Nfe2l2	**NFE2 Like BZIP Transcription Factor 2 (Nrf2)**	ATTGCCACCGCCAGGACTA	TGCCTCCAAAGGATGTCAATCAA
Cox2a/Ptgs2	**Prostaglandin-Endoperoxide Synthase 2 (Cox2)**	AGATCAGAAGCGAGGACCT	CCATCCTGGAAAAGTCGAA
Ptgr1	**Prostaglandin Reductase 1**	TACCACTGTCTTTGGCTTTGGG	CCCTTCAAGCCACAGATGTCAA
Gclc	**Glutamate-Cysteine Ligase Catalytic Subunit**	GTCTTCAGGTGACATTCCAAGC	TGTTCTTCAGGGGCTCCAGTC
Gclm	**Glutamate-Cysteine Ligase Modifier Subunit**	CTGCTAAACTGTTCATTGTAGG	CTATTGGGTTTTACCTGTG
Gsr	**Glutathione-Disulfide Reductase**	GAAGATGGTTTGTGCCAACAAAGAG	GCATATGGATGCCAACCACCTT
Gsta4	**Glutathione S-Transferase Alpha 4**	GAAGTTCTAGTGACAGCGTGCTTTA	TGTAGCTGCTGCTGATTGG
Ppar-g	**Peroxisome Proliferator Activated Receptor Gamma**	TCAGGTTTGGGCGAATG	TTTGGTCAGCGGGAAGG
Cldn1	**Claudin 1**	TCGACTCCTTGCTGAATCTG	ACTTCATGCCAATGGTGGAC
Occl	**Occludin**	CATCTGACTATGCGGAAAG	GGTGTCTCTAGGTTATCGT
ZO1	**Zonula Occludens/Tight junction protein 2**	GATCATTCCACACAGTCTCC	CACTGCTTTGGGTGTATGAT
INF-γ	**Interferon gamma**	TGAGCATCGCCAAGTTCGAG	CACCGACTCCTTTTCCGCTTC
TNF-α	Tumour necrosis factor alpha	AAATGGGCTCCCTCTCATCAGTTC	TCTGCTTGGTGGTTTGCTACGAC
IL-6	**Interleukin 6**	TCCTACCCCAACTTCCAATGCTC	TTGGATGGTCTTGGTCCTTAGCC
IL-10	**Interleukin 10**	GCAGTGGAGCAGGTGAAGAATG	CAGTCCAGTAGATGCCGGGT
IL-12	**Interleukin 12**	GCATGTGTCAATCACGCTACC	GTATGACCCTGGCCGAAGTG
IL-15	**Interleukin 15**	TGTGTCAGTGTAGGTCTCCCT	TGCAGTAACTTTGCAACTGGG
IL-18	**Interleukin 18**	CAAAAGAAACCCGCCTGTGTT	AGTCTGGTCTGGGATTCGTTG
Polr2a	**RNA Polymerase II Subunit A**	CGGCGTCCTGAGTCCG	AACTTGGGGGACTAATGGATCC

Table S4: Bacterial taxonomy of species found in faecal microbiota of rats

Phylum	Class	Order	Family	Genus	Species
Actinobacteriota	*Actinobacteria*	*Bifidobacteriales*	*Bifidobacteriaceae*	*Bifidobacterium*	*Bifidobacterium animalis*
Actinobacteriota	*Actinobacteria*	*Bifidobacteriales*	*Bifidobacteriaceae*	*Bifidobacterium*	*Bifidobacterium pseudolongum*
Actinobacteriota	*Actinobacteria*	*Corynebacteriales*	*Corynebacteriaceae*	*Corynebacterium*	*Corynebacterium lowii*
Actinobacteriota	*Actinobacteria*	*Micrococcales*	*Micrococcaceae*	*Rothia*	*Rothia nasimurium*
Actinobacteriota	*Coriobacteriia*	*Coriobacteriales*	*Atopobiaceae*	*Coriobacteriaceae* UCG-002	unknown species
Actinobacteriota	*Coriobacteriia*	*Coriobacteriales*	*Eggerthellaceae*	*Adlercreutzia*	*Adlercreutzia equolifaciens*
Actinobacteriota	*Coriobacteriia*	*Coriobacteriales*	*Eggerthellaceae*	*Adlercreutzia*	unknown species
Actinobacteriota	*Coriobacteriia*	*Coriobacteriales*	*Eggerthellaceae*	*Enterorhabdus*	*Adlercreutzia mucosicola*
Actinobacteriota	*Coriobacteriia*	*Coriobacteriales*	*Eggerthellaceae*	*Enterorhabdus*	unknown species
Actinobacteriota	*Coriobacteriia*	*Coriobacteriales*	*Eggerthellaceae*	*Gordonibacter*	unknown species
Actinobacteriota	*Coriobacteriia*	*Coriobacteriales*	*Eggerthellaceae*	*Parvibacter*	unknown species
Bacteroidota	*Bacteroidia*	*Bacteroidales*	*Bacteroidaceae*	*Bacteroides*	*Bacteroides acidifaciens*
Bacteroidota	*Bacteroidia*	*Bacteroidales*	*Bacteroidaceae*	*Bacteroides*	*Bacteroides caccae*
Bacteroidota	*Bacteroidia*	*Bacteroidales*	*Bacteroidaceae*	*Bacteroides*	*Bacteroides dorei*
Bacteroidota	*Bacteroidia*	*Bacteroidales*	*Bacteroidaceae*	*Bacteroides*	*Bacteroides massiliensis*
Bacteroidota	*Bacteroidia*	*Bacteroidales*	*Bacteroidaceae*	*Bacteroides*	*Bacteroides thetaiotaomicron*
Bacteroidota	*Bacteroidia*	*Bacteroidales*	*Bacteroidaceae*	*Bacteroides*	*Bacteroides vulgatus*
Bacteroidota	*Bacteroidia*	*Bacteroidales*	*Bacteroidaceae*	*Bacteroides*	*Bacteroides xylanisolvens*
Bacteroidota	*Bacteroidia*	*Bacteroidales*	*Bacteroidaceae*	*Bacteroides*	unknown species
Bacteroidota	*Bacteroidia*	*Bacteroidales*	*Marinifilaceae*	*Butyricimonas*	unknown species
Bacteroidota	*Bacteroidia*	*Bacteroidales*	*Marinifilaceae*	*Odoribacter*	unknown species
Bacteroidota	*Bacteroidia*	*Bacteroidales*	*Muribaculaceae*	*Muribaculum*	*Muribaculum intestinale*
Bacteroidota	*Bacteroidia*	*Bacteroidales*	*Muribaculaceae*	unknown genus	unknown species
Bacteroidota	*Bacteroidia*	*Bacteroidales*	*Prevotellaceae*	*Alloprevotella*	unknown species
Bacteroidota	*Bacteroidia*	*Bacteroidales*	*Rikenellaceae*	*Alistipes*	*Alistipes finegoldii*
Bacteroidota	*Bacteroidia*	*Bacteroidales*	*Rikenellaceae*	*Alistipes*	*Alistipes indistinctus*
Bacteroidota	*Bacteroidia*	*Bacteroidales*	*Rikenellaceae*	*Alistipes*	*Alistipes timonensis*
Bacteroidota	*Bacteroidia*	*Bacteroidales*	*Rikenellaceae*	*Alistipes*	unknown species
Bacteroidota	*Bacteroidia*	*Bacteroidales*	*Rikenellaceae*	*Rikenella*	unknown species
Bacteroidota	*Bacteroidia*	*Bacteroidales*	*Rikenellaceae*	*Rikenellaceae* RC9 gut group	unknown species
Bacteroidota	*Bacteroidia*	*Bacteroidales*	*Tannerellaceae*	*Parabacteroides*	*Parabacteroides distasonis*
Bacteroidota	*Bacteroidia*	*Bacteroidales*	*Tannerellaceae*	*Parabacteroides*	*Parabacteroides goldsteinii*
Bacteroidota	*Bacteroidia*	*Bacteroidales*	*Tannerellaceae*	*Parabacteroides*	*Parabacteroides merdae*
Bacteroidota	*Bacteroidia*	*Bacteroidales*	*Tannerellaceae*	*Parabacteroides*	unknown species
Deferribacterota	*Deferribacteres*	*Deferribacterales*	*Deferribacteraceae*	*Mucispirillum*	*Mucispirillum schaedleri*
Desulfobacterota	*Desulfovibrionia*	*Desulfovibrionales*	*Desulfovibrionaceae*	*Bilophila*	unknown species
Desulfobacterota	*Desulfovibrionia*	*Desulfovibrionales*	*Desulfovibrionaceae*	*Desulfovibrio*	unknown species
Desulfobacterota	*Desulfovibrionia*	*Desulfovibrionales*	*Desulfovibrionaceae*	unknown genus	unknown species
Firmicutes	*Bacilli*	*Bacillales*	*Bacillaceae*	*Bacillus*	*Bacillus mycoides*
Firmicutes	*Bacilli*	*Erysipelotrichales*	*Erysipelatoclostridiaceae*	*Candidatus Stoquefichus*	*Candidatus Stoquefichus massiliensis*
Firmicutes	*Bacilli*	*Erysipelotrichales*	*Erysipelatoclostridiaceae*	*Erysipelatoclostridium*	*Erysipelatoclostridium ramosum*
Firmicutes	*Bacilli*	*Erysipelotrichales*	*Erysipelatoclostridiaceae*	*Erysipelatoclostridium*	unknown species
Firmicutes	*Bacilli*	*Erysipelotrichales*	*Erysipelatoclostridiaceae*	unknown genus	unknown species
Firmicutes	*Bacilli*	*Erysipelotrichales*	*Erysipelotrichaceae*	*Holdemania*	*Holdemania filiformis*
Firmicutes	*Bacilli*	*Erysipelotrichales*	*Erysipelotrichaceae*	*Turicibacter*	unknown species
Firmicutes	*Bacilli*	*Exiguobacterales*	*Exiguobacteraceae*	*Exiguobacterium*	Multi-affiliation
Firmicutes	*Bacilli*	*Lactobacillales*	*Enterococcaceae*	*Enterococcus*	*Enterococcus faecalis*
Firmicutes	*Bacilli*	*Lactobacillales*	*Enterococcaceae*	*Enterococcus*	Multi-affiliation
Firmicutes	*Bacilli*	*Lactobacillales*	*Lactobacillaceae*	*Lactobacillus*	*Lactobacillus hominis*
Firmicutes	*Bacilli*	*Lactobacillales*	*Lactobacillaceae*	*Lactobacillus*	*Lactobacillus intestinalis*
Firmicutes	*Bacilli*	*Lactobacillales*	*Lactobacillaceae*	*Lactobacillus*	*Lactobacillus reuteri*
Firmicutes	*Bacilli*	*Lactobacillales*	*Lactobacillaceae*	*Lactobacillus*	Multi-affiliation
Firmicutes	*Bacilli*	*Lactobacillales*	*Lactobacillaceae*	*Lactobacillus*	unknown species
Firmicutes	*Bacilli*	*Lactobacillales*	*Streptococcaceae*	*Lactococcus*	unknown species
Firmicutes	*Bacilli*	*Lactobacillales*	*Streptococcaceae*	*Streptococcus*	*Streptococcus azizii*
Firmicutes	*Bacilli*	*Lactobacillales*	*Streptococcaceae*	*Streptococcus*	unknown species
Firmicutes	*Bacilli*	*Staphylococcales*	*Staphylococcaceae*	*Macrococcus*	Multi-affiliation
Firmicutes	*Bacilli*	*Staphylococcales*	*Staphylococcaceae*	*Staphylococcus*	Multi-affiliation
Firmicutes	*Clostridia*	*Christensenellales*	*Christensenellaceae*	*Christensenellaceae* R-7 group	unknown species
Firmicutes	*Clostridia*	*Christensenellales*	*Christensenellaceae*	unknown genus	unknown species
Firmicutes	*Clostridia*	*Clostridia UCG-014*	unknown family	unknown genus	unknown species
Firmicutes	*Clostridia*	*Clostridia vadinBB60 group*	unknown family	unknown genus	unknown species
Firmicutes	*Clostridia*	*Clostridiales*	*Clostridiaceae*	*Clostridium sensu stricto* 1	unknown species
Firmicutes	*Clostridia*	*Clostridiales*	*Hungateiclostridiaceae*	*Ruminiclostridium*	unknown species
Firmicutes	*Clostridia*	*Eubacteriales*	*Anaerofustaceae*	*Anaerofustis*	unknown species
Firmicutes	*Clostridia*	*Eubacteriales*	*Eubacteriaceae*	*Eubacterium*	Multi-affiliation
Firmicutes	*Clostridia*	*Lachnospirales*	*Defluviitaleaceae*	*Defluviitaleaceae* UCG-011	unknown species
Firmicutes	*Clostridia*	*Lachnospirales*	*Lachnospiraceae*	*[Eubacterium] xylanophilum* group	unknown species
Firmicutes	*Clostridia*	*Lachnospirales*	*Lachnospiraceae*	*[Ruminococcus] gauvreauii* group	*Ruminococcus gauvreauii*
Firmicutes	*Clostridia*	*Lachnospirales*	*Lachnospiraceae*	*[Ruminococcus] gauvreauii* group	unknown species
Firmicutes	*Clostridia*	*Lachnospirales*	*Lachnospiraceae*	*[Ruminococcus] torques* group	unknown species
Firmicutes	*Clostridia*	*Lachnospirales*	*Lachnospiraceae*	A2	unknown species
Firmicutes	*Clostridia*	*Lachnospirales*	*Lachnospiraceae*	*Acetatifactor*	unknown species
Firmicutes	*Clostridia*	*Lachnospirales*	*Lachnospiraceae*	*Agathobacter*	unknown species
Firmicutes	*Clostridia*	*Lachnospirales*	*Lachnospiraceae*	*Anaerostipes*	unknown species
Firmicutes	*Clostridia*	*Lachnospirales*	*Lachnospiraceae*	ASF356	unknown species
Firmicutes	*Clostridia*	*Lachnospirales*	*Lachnospiraceae*	*Blautia*	*Blautia glucerasea*
Firmicutes	*Clostridia*	*Lachnospirales*	*Lachnospiraceae*	*Blautia*	Multi-affiliation
Firmicutes	*Clostridia*	*Lachnospirales*	*Lachnospiraceae*	*Blautia*	unknown species
Firmicutes	*Clostridia*	*Lachnospirales*	*Lachnospiraceae*	*Butyrivibrio*	unknown species
Firmicutes	*Clostridia*	*Lachnospirales*	*Lachnospiraceae*	*Coprococcus*	unknown species
Firmicutes	*Clostridia*	*Lachnospirales*	*Lachnospiraceae*	*Dorea*	unknown species
Firmicutes	*Clostridia*	*Lachnospirales*	*Lachnospiraceae*	*Eisenbergiella*	unknown species
Firmicutes	*Clostridia*	*Lachnospirales*	*Lachnospiraceae*	*Frisingicoccus*	*Frisingicoccus caecimuris*
Firmicutes	*Clostridia*	*Lachnospirales*	*Lachnospiraceae*	GCA-900066575	unknown species
Firmicutes	*Clostridia*	*Lachnospirales*	*Lachnospiraceae*	*Hungatella*	*Hungatella hathewayi*
Firmicutes	*Clostridia*	*Lachnospirales*	*Lachnospiraceae*	*Lachnoclostridium*	Multi-affiliation
Firmicutes	*Clostridia*	*Lachnospirales*	*Lachnospiraceae*	*Lachnoclostridium*	unknown species
Firmicutes	*Clostridia*	*Lachnospirales*	*Lachnospiraceae*	*Lachnospiraceae* FCS020 group	unknown species
Firmicutes	*Clostridia*	*Lachnospirales*	*Lachnospiraceae*	*Lachnospiraceae* NK4A136 group	unknown species
Firmicutes	*Clostridia*	*Lachnospirales*	*Lachnospiraceae*	*Lachnospiraceae* UCG-001	unknown species
Firmicutes	*Clostridia*	*Lachnospirales*	*Lachnospiraceae*	*Lachnospiraceae* UCG-006	unknown species
Firmicutes	*Clostridia*	*Lachnospirales*	*Lachnospiraceae*	*Lachnospiraceae* UCG-008	unknown species
Firmicutes	*Clostridia*	*Lachnospirales*	*Lachnospiraceae*	*Marvinbryantia*	unknown species
Firmicutes	*Clostridia*	*Lachnospirales*	*Lachnospiraceae*	Multi-affiliation	Multi-affiliation
Firmicutes	*Clostridia*	*Lachnospirales*	*Lachnospiraceae*	*Roseburia*	unknown species
Firmicutes	*Clostridia*	*Lachnospirales*	*Lachnospiraceae*	*Sellimonas*	unknown species
Firmicutes	*Clostridia*	*Lachnospirales*	*Lachnospiraceae*	*Tuzzerella*	unknown species
Firmicutes	*Clostridia*	*Lachnospirales*	*Lachnospiraceae*	*Tyzzerella*	*[Clostridium] colinum*
Firmicutes	*Clostridia*	*Lachnospirales*	*Lachnospiraceae*	*unknown genus*	unknown species
Firmicutes	*Clostridia*	*Monoglobales*	*Monoglobaceae*	*Monoglobus*	unknown species
Firmicutes	*Clostridia*	*Oscillospirales*	*[Clostridium] methylpentosum* group	unknown genus	*[Clostridium] methylpentosum*
Firmicutes	*Clostridia*	*Oscillospirales*	*[Eubacterium] coprostanoligenes* group	unknown genus	unknown species
Firmicutes	*Clostridia*	*Oscillospirales*	*Butyricicoccaceae*	*UCG-009*	unknown species
Firmicutes	*Clostridia*	*Oscillospirales*	*Ethanoligenenaceae*	*Acetanaerobacterium*	unknown species
Firmicutes	*Clostridia*	*Oscillospirales*	Multi-affiliation	Multi-affiliation	Multi-affiliation
Firmicutes	*Clostridia*	*Oscillospirales*	*Oscillospiraceae*	*Colidextribacter*	unknown species
Firmicutes	*Clostridia*	*Oscillospirales*	*Oscillospiraceae*	*Flavonifractor*	*Flavonifractor plautii*
Firmicutes	*Clostridia*	*Oscillospirales*	*Oscillospiraceae*	*Intestinimonas*	*Intestinimonas butyriciproducens*
Firmicutes	*Clostridia*	*Oscillospirales*	*Oscillospiraceae*	*Intestinimonas*	unknown species
Firmicutes	*Clostridia*	*Oscillospirales*	*Oscillospiraceae*	Multi-affiliation	Multi-affiliation
Firmicutes	*Clostridia*	*Oscillospirales*	*Oscillospiraceae*	NK4A214 group	unknown species
Firmicutes	*Clostridia*	*Oscillospirales*	*Oscillospiraceae*	*Oscillibacter*	unknown species
Firmicutes	*Clostridia*	*Oscillospirales*	*Oscillospiraceae*	*Oscillospira*	unknown species
Firmicutes	*Clostridia*	*Oscillospirales*	*Oscillospiraceae*	UCG-003	unknown species
Firmicutes	*Clostridia*	*Oscillospirales*	*Oscillospiraceae*	UCG-005	unknown species
Firmicutes	*Clostridia*	*Oscillospirales*	*Oscillospiraceae*	unknown genus	unknown species
Firmicutes	*Clostridia*	*Oscillospirales*	*Ruminococcaceae*	*[Eubacterium] siraeum group*	unknown species
Firmicutes	*Clostridia*	*Oscillospirales*	*Ruminococcaceae*	*Anaerofilum*	unknown species
Firmicutes	*Clostridia*	*Oscillospirales*	*Ruminococcaceae*	*Anaerotruncus*	unknown species
Firmicutes	*Clostridia*	*Oscillospirales*	*Ruminococcaceae*	*Candidatus Soleaferrea*	*Candidatus Soleaferrea massiliensis*
Firmicutes	*Clostridia*	*Oscillospirales*	*Ruminococcaceae*	*Candidatus Soleaferrea*	unknown species
Firmicutes	*Clostridia*	*Oscillospirales*	*Ruminococcaceae*	*Faecalibacterium*	unknown species
Firmicutes	*Clostridia*	*Oscillospirales*	*Ruminococcaceae*	*Harryflintia*	unknown species
Firmicutes	*Clostridia*	*Oscillospirales*	*Ruminococcaceae*	Multi-affiliation	Multi-affiliation
Firmicutes	*Clostridia*	*Oscillospirales*	*Ruminococcaceae*	*Negativibacillus*	unknown species
Firmicutes	*Clostridia*	*Oscillospirales*	*Ruminococcaceae*	*Pygmaiobacter*	unknown species
Firmicutes	*Clostridia*	*Oscillospirales*	*Ruminococcaceae*	*Ruminococcus*	unknown species
Firmicutes	*Clostridia*	*Oscillospirales*	*Ruminococcaceae*	unknown genus	unknown species
Firmicutes	*Clostridia*	*Oscillospirales*	UCG-010	unknown genus	unknown species
Firmicutes	*Clostridia*	*Peptococcales*	*Peptococcaceae*	*Peptococcus*	unknown species
Firmicutes	*Clostridia*	*Peptococcales*	*Peptococcaceae*	unknown genus	unknown species
Firmicutes	*Clostridia*	*Peptostreptococcales-Tissierellales*	*Anaerovoracaceae*	*[Eubacterium] brachy* group	unknown species
Firmicutes	*Clostridia*	*Peptostreptococcales-Tissierellales*	*Anaerovoracaceae*	*[Eubacterium] nodatum* group	unknown species
Firmicutes	*Clostridia*	*Peptostreptococcales-Tissierellales*	*Anaerovoracaceae*	*Anaerovorax*	unknown species
Firmicutes	*Clostridia*	*Peptostreptococcales-Tissierellales*	*Anaerovoracaceae*	Family XIII AD3011 group	unknown species
Firmicutes	*Clostridia*	*Peptostreptococcales-Tissierellales*	*Peptostreptococcaceae*	*Romboutsia*	*Romboutsia ilealis*
Firmicutes	*Negativicutes*	*Acidaminococcales*	*Acidaminococcaceae*	*Phascolarctobacterium*	*Phascolarctobacterium succinatutens*
Firmicutes	*Negativicutes*	*Acidaminococcales*	*Acidaminococcaceae*	*Phascolarctobacterium*	unknown species
Proteobacteria	*Alphaproteobacteria*	*Rhodospirillales*	unknown family	unknown genus	unknown species
Proteobacteria	*Gammaproteobacteria*	*Burkholderiales*	*Sutterellaceae*	*Parasutterella*	*Parasutterella excrementihominis*
Proteobacteria	*Gammaproteobacteria*	*Burkholderiales*	*Sutterellaceae*	*Parasutterella*	unknown species
Proteobacteria	*Gammaproteobacteria*	*Enterobacterales*	*Enterobacteriaceae*	*Escherichia-Shigella*	*Escherichia coli*
Proteobacteria	*Gammaproteobacteria*	*Enterobacterales*	*Enterobacteriaceae*	*Escherichia-Shigella*	unknown species

Supplementary Figures

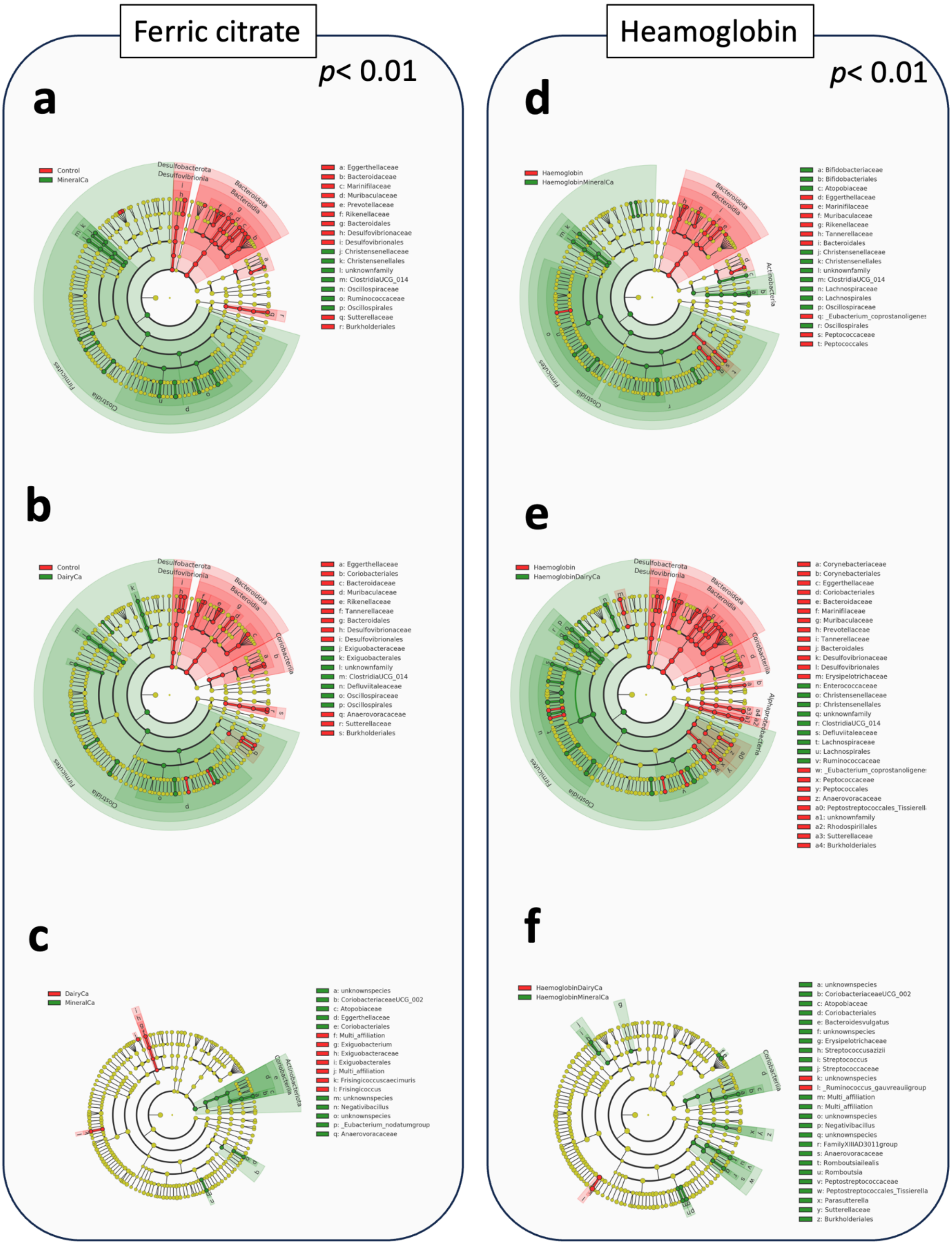

Fig. S1: Impact of dietary calcium content (Control *vs.* Mineral *vs.* Dairy Calcium) on abundance of faecal bacterial taxa in rats fed ferric citrate or haemoglobin.

Circular cladogram generated from LEfSe analysis showing the most differentially abundant taxa enriched in microbiota from rats fed with:

- **(a)** Control (red) or Mineral Calcium (green)
- **(b)** Control (red) or Dairy Calcium (green)
- **(c)** Mineral Calcium (green) or Dairy Calcium (red)
- **(d)** Haemoglobin (red) or Haemoglobin supplemented with Mineral Calcium (green)
- **(e)** Haemoglobin (red) or Haemoglobin supplemented with Dairy Calcium (green)
- **(f)** Haemoglobin supplemented with Mineral (green) or Dairy Calcium (red).

Only features with **LDA scores >3,** a ***p*-value <0.01** (Kruskal-Wallis sum-rank test) and that pass the threshold of ***q*-value <0.05** using pairwise tests among the sub-group (Wilcoxon rank-sum test), were plotted. Corresponding LDA scores are provided in archive entitled "W2_Calcium" at https://entrepot.recherche.data.gouv.fr (https://doi.org/10.57745/XYPYPY).

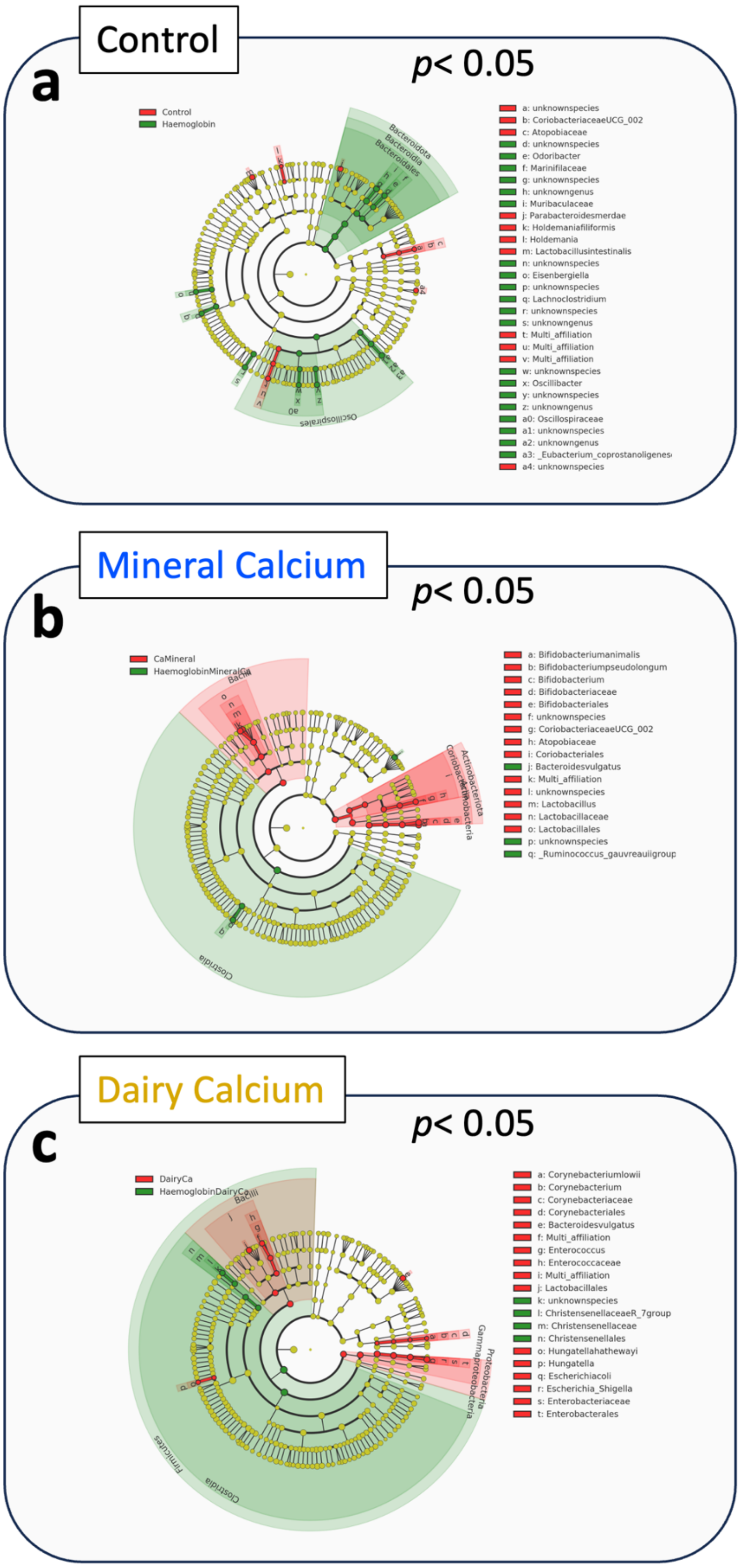

Fig. S2: Impact of dietary iron form (Ferric citrate *vs.* Haemoglobin) on abundance of faecal bacterial taxa in rats fed variable calcium intake.

Circular cladogram generated from LEfSe analysis showing the most differentially abundant taxa enriched in microbiota from rats fed with:

(a) Control (i.e, ferric citrate in red) or Haemoglobin (green).

(b) Mineral Calcium (red) or Haemoglobin supplemented with Mineral Calcium (green).

(c) Dairy Calcium (red) or Haemoglobin supplemented with Dairy Calcium (green).

Only features with **LDA scores >3,** a ***p*-value <0.05** (Kruskal-Wallis sum-rank test) and that pass the threshold of ***q*-value <0.05** using pairwise tests among the sub-group (Wilcoxon rank-sum test), were plotted. Corresponding LDA scores are provided in archive entitled "W2_Calcium" at https://entrepot.recherche.data.gouv.fr (https://doi.org/10.57745/XYPYPY).

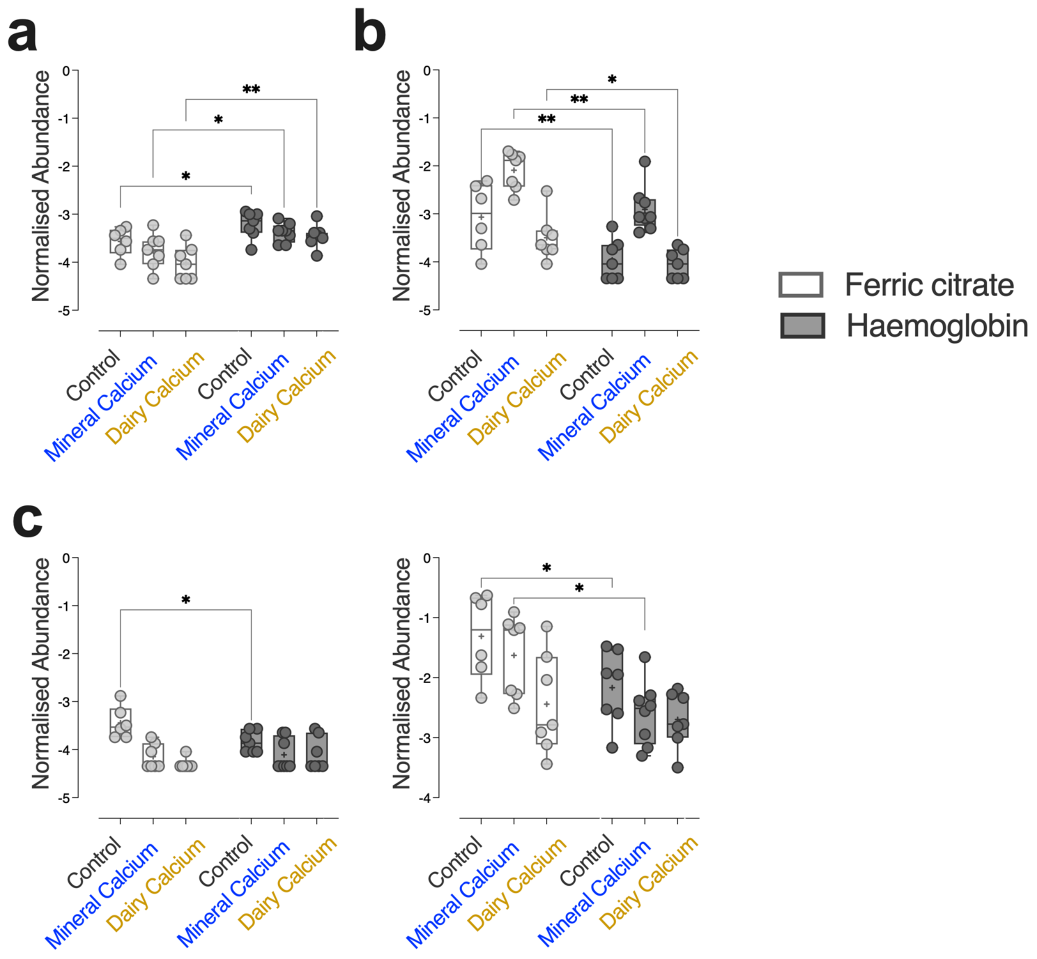

Fig. S3: Haemoglobin-induced changes in faecal microbiota not normalised by the addition of dietary calcium, regardless of its origin. Alteration of the relative abundances of some bacterial communities by dietary haemoglobin (agglomeration of OTUs at the species level):

> **(a)** was not normalised by calcium intake (*Butyrococcus* UCG-009). No impact of calcium was observed.
>
> **(b)** was modulated by the calcium intake but did not normalise to levels observed with ferric citrate (*Coriobacteriaceae* UCG-002).
>
> **(c)** was reinforced by dietary calcium regardless of its source (*Candidatus Soleaferrea massiliensis,* or unknown *Lactobacillus*)*.* They were indeed affected by calcium, even without the addition of haemoglobin.

Individual normalised values were plotted within box and whisker with "+" as means according to dietary iron (Ferric citrate *vs.* Haemoglobin) and calcium (Control *vs.* Mineral *vs.* Dairy) contents. For statistical analysis, two-way ANOVA, followed by Holm-Šídák's multiple comparisons tests were performed. *p_{adj} <0.05, **p_{adj}<0.01 according to dietary iron form. Detailed F-statistic and *p* values are provided as supporting information (Table S1).

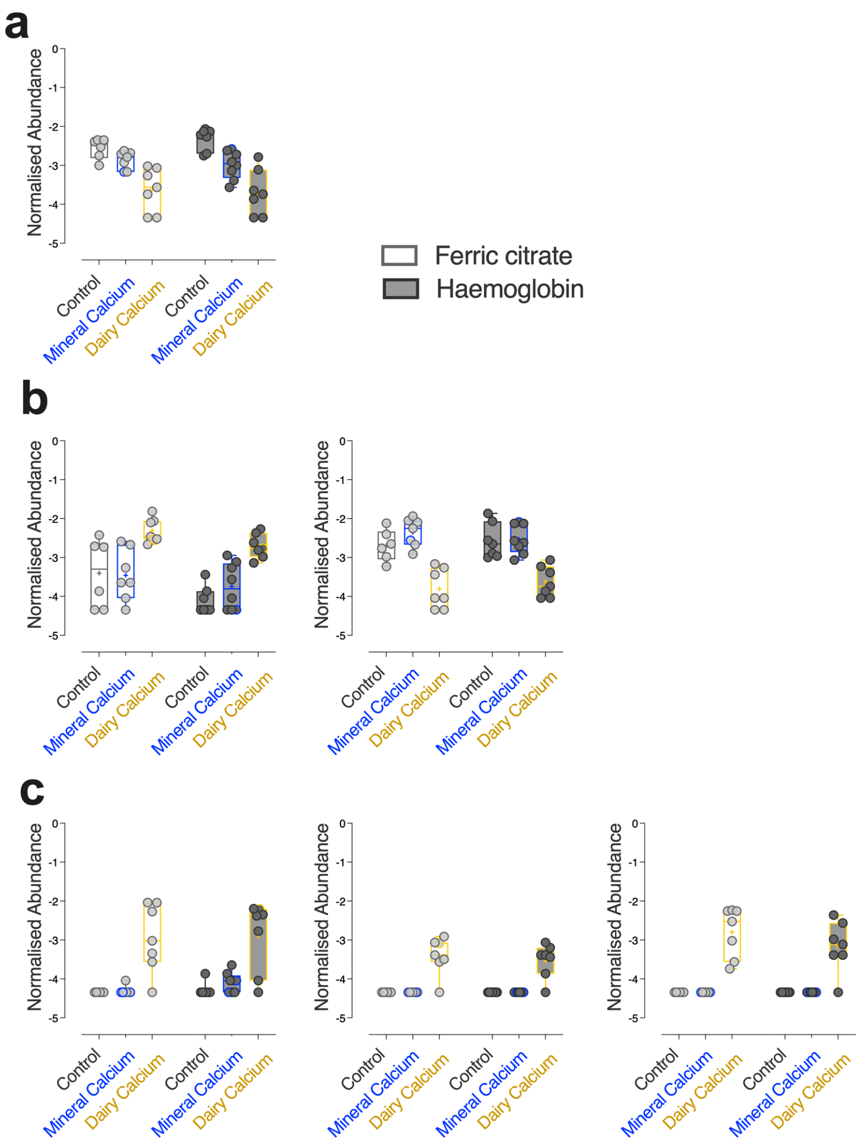

Fig. S4: Bacterial communities not affected by dietary haemoglobin but differentially influenced by dietary calcium. Among bacterial communities that were unaffected by dietary haemoglobin (agglomeration of OTUs at the species level):

> **(a)** The faecal relative abundance of *Desulfovibrio spp*. was diminished by calcium supplementation, and especially when calcium was provided by the dairy matrix.
>
> **(b)** The faecal relative abundances of some bacterial communities were specifically modulated by dietary calcium when it was provided by the dairy matrix and not in mineral form: multi-affiliated *Enterococcus*, *Negativibacillus spp*.
>
> **(c)** Some bacterial communities were exclusively found in faeces of rats fed the diet supplemented with dairy calcium: *Frisingicoccus caecimuris*, *Macrococcus spp*., *Exiguobacterium spp.*

Individual normalised values were plotted within box and whisker with "+" as means according to dietary iron (Ferric citrate *vs.* Haemoglobin) and calcium (Control *vs.* Mineral *vs.* Dairy) contents. For statistical analysis, a two-way ANOVA was performed. Detailed F-statistic and *p* values are provided as supporting information (Table S1).
